# Vibrational exciton delocalization precludes the use of infrared intensities as proxies for surfactant accumulation on aqueous surfaces†

**DOI:** 10.1039/d1sc01276b

**Published:** 2021-05-18

**Authors:** Kimberly A. Carter-Fenk, Kay Carter-Fenk, Michelle E. Fiamingo, Heather C. Allen, John M. Herbert

**Affiliations:** a Department of Chemistry & Biochemistry, The Ohio State University Columbus OH USA allen@chemistry.ohio-state.edu herbert@chemistry.ohio-state.edu

## Abstract

Surface-sensitive vibrational spectroscopy is a common tool for measuring molecular organization and intermolecular interactions at interfaces. Peak intensity ratios are typically used to extract molecular information from one-dimensional spectra but vibrational coupling between surfactant molecules can manifest as signal depletion in one-dimensional spectra. Through a combination of experiment and theory, we demonstrate the emergence of vibrational exciton delocalization in infrared reflection–absorption spectra of soluble and insoluble surfactants at the air/water interface. Vibrational coupling causes a significant decrease in peak intensities corresponding to C–F vibrational modes of perfluorooctanoic acid molecules. Vibrational excitons also form between arachidic acid surfactants within a compressed monolayer, manifesting as signal reduction of C–H stretching modes. Ionic composition of the aqueous phase impacts surfactant intermolecular distance, thereby modulating vibrational coupling strength between surfactants. Our results serve as a cautionary tale against employing alkyl and fluoroalkyl vibrational peak intensities as proxies for concentration, although such analysis is ubiquitous in interface science.

## Abstract 2

Coupling between surfactant molecules at the air/water interface bleeds intensity into a diffuse background, such that single-wavelength vibrational intensity is effectively depleted at high surface coverage.

## Graphical abstract

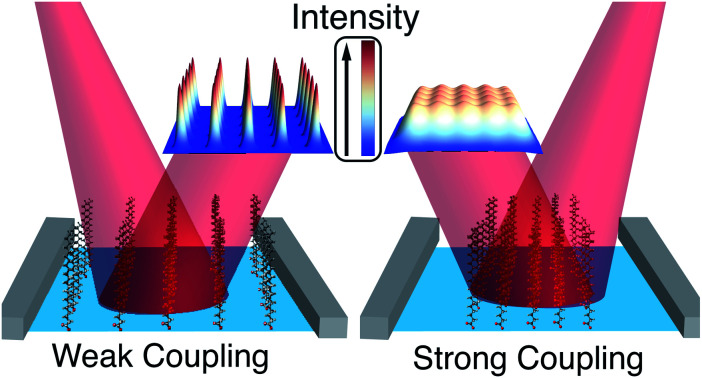

## 1 Introduction

Elucidating structure at the air/water interface is important to understanding chemical reactivity in biological, environmental, and industrial systems. Surfactants comprise an abundant class of amphiphilic molecules that partition at the air/water interface with vibrational modes that are typically well-separated from those of water, offering a sensitive probe of interfacial adsorption and organization *via* vibrational spectroscopy.^1^ Moreover, surfactant molecules spontaneously aggregate into micelles at high concentrations,^2^ and strong vibrational couplings play a central role in the spectra of self-aggregating nanostructures.^3^ In the present work, we apply vibrational exciton theory^4,5^ to understand the role of vibrational coupling between surfactant molecules at the air/water interface. In this approach, which is the vibrational analogue of the Frenkel–Davydov exciton theory developed to understand collective electronic excitations in molecular crystals,^6,7^ the vibrational wavefunction is expressed as a superposition of local-mode wavefunctions on individual vibrational chromophores. Collective vibrational “excitons” emerge from coupling between quasi-degenerate local modes, and these have the effect of depleting local signal intensity in one-dimensional (1D) spectroscopic measurements.^3,8–11^ Total oscillator strength (when integrated over all wavelengths) is conserved in the presence of excitonic coupling, and the apparent loss in signal at a particular wavelength is a result of intensity borrowing leading to a depletion of oscillator strength from the nominal bright state(s). In the presence of significant coupling, much of the oscillator strength may be “lost” in a diffuse background that is difficult to resolve experimentally. This can lead to misinterpretation of the results of 1D-IR experiments. The effects of exciton coupling on 1D-IR spectra have been well documented in molecular clusters,^8^ liquid water,^12–15^ proteins,^16–18^ DNA,^19,20^ and for molecules adsorbed on solid surfaces,^21–23^ but the influence of these couplings at the air/water interface has yet to be explored.

In excitonic spectra, vibrational coupling splits the parent frequencies *ω*_0_ into pairs of frequencies *ω*_+_ and *ω*_−_ for each pair of interacting chromophores. The resulting spectrum is more delocalized in frequency and the oscillator strength is distributed unevenly across the new modes *ω*_±_, many of which feature near-zero intensities. The concomitant intensity borrowing can cause oscillator strength to be sapped from the nominal bright states (frequencies *ω*_0_), which are “dissolved” in a bath of emergent low-intensity peaks.^24^ Operationally, this can manifest as a dramatic reduction in the experimentally observable oscillator strength (even though total oscillator strength is conserved), as many of the emergent modes may lie well below detection limits.

Two-dimensional (2D) vibrational spectroscopic techniques are the only methods immune to this problem because they probe vibrational coupling directly, although the resulting spectra are sometimes too convoluted to analyze. To date, only three studies have investigated vibrational coupling between alkyl chains of a surfactant monolayer.^25–27^ Bredenbeck *et al.*^25,26^ first observed vibrational coupling between the terminal methyl and methylene groups of a dodecanol monolayer at the air/water interface using 2D vibrational sum frequency generation spectroscopy (VSFG). A wealth of structural information is present within the off-diagonal C–H peaks in 2D vibrational spectra, but accessing many of these details can be unintuitive.

One-dimensional vibrational spectroscopic techniques such as surface-sensitive infrared reflection–absorption spectroscopy (IRRAS) and surface-selective VSFG are simpler to dissect than their 2D analogues and have been much more widely adopted for the analysis of surfactant organization at interfaces. Soluble surfactant adsorption and film surface density are measured through vibrational band peak intensities in which hydrocarbon C–H and C–D modes are most commonly tracked.^28–32^ The orientation of surfactant terminal methyl groups is frequently assessed *via* 1D VSFG and by calculating ratios of CH_3_ peak intensities obtained by probing the system with different polarizations of light.^33,34^ An analogous analysis using the CH_2_ peak intensities from 1D IRRAS spectra is used to assess hydrocarbon chain conformational order at interfaces.^35–38^ A method was recently developed to estimate the total intermolecular interactions in a surfactant monolayer at the air/water interface using the intensity ratio amplitude of the second-order Fermi resonance signals generated by VSFG.^39^ All of the above analyses rely upon alkyl chain peak intensities, which are susceptible to signal strength reduction *via* vibrational coupling.

To investigate the influence of vibrational excitons on 1D vibrational spectra, we collected IRRAS spectra and surface tension measurements of perfluorooctanoic acid (PFOA) at the air/water interface and compared the experimental data to theoretical spectra generated from molecular dynamics (MD) simulations combined with an *ab initio* vibrational exciton model (*ai*VEM). Both theory and experiment reveal signal intensity depletion in the C–F vibrational modes at PFOA concentrations near but below the critical micelle concentration. Signal depletion due to vibrational excitons is also observed in the C–H peaks of an insoluble arachidic acid monolayer upon lateral compression, suggesting that the formation of vibrational excitons is a general feature of 1D vibrational spectra of surfactants at interfaces. This study therefore serves as a cautionary tale against structural analyses using alkyl- and fluoroalkyl-chain vibrational mode peak intensities.

## 2 Results and discussion

### PFOA adsorption measurements

Vibrational exciton formation in soluble surfactant films at the air/aqueous interface of ultrapure H_2_O and 0.47 M NaCl solution was studied with surface-sensitive IRRAS. The specific concentration of NaCl was chosen to match the Na^+^ concentration in seawater, and the soluble surfactant PFOA [CF_3_(CF_2_)_6_COOH] was selected due to its surface activity and large perfluoroalkyl transition dipole moments. Additionally, PFOA is a strong acid (p*K*_a_ < 1 by recent estimates),^40,41^ so the surfactant is fully deprotonated in the bulk and at the surface of ultrapure water (pH = 5.8). IRRAS spectra are analyzed in the C–F region (∼1100–1300 cm^−1^) and the spectra are plotted as reflectance–absorbance (RA),1
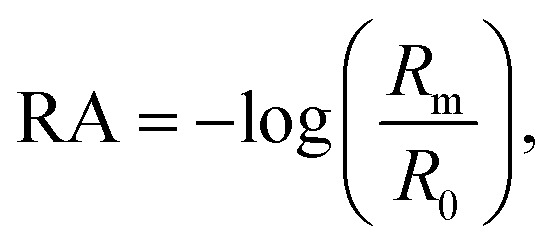
where *R*_m_ is the reflectivity of the PFOA film and *R*_0_ is the reflectivity of the aqueous subphase without PFOA.

**Fig. 1:**
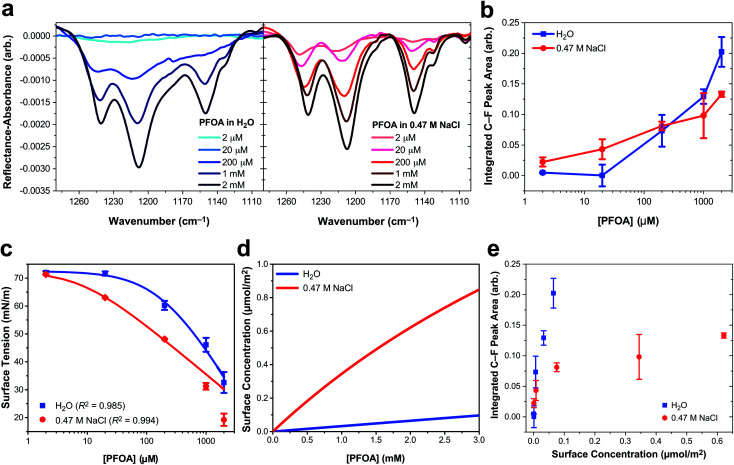
Perfluorooctanoic acid (PFOA) adsorption to the water and 0.47 M NaCl(aq) interface. (a) Infrared reflection–absorption spectroscopy (IRRAS) of the C–F vibrational mode region as a function of PFOA bulk concentration. (b) Integrated peak area corresponding to the C–F vibrational modes as a function of the bulk PFOA concentration. Lines connecting the data points are drawn to guide the eye. (c) Surface tension measurements as a function of PFOA bulk concentration. The curved lines connecting the data are fits to the Szyszkowski equation [eqn (2)] and the adjusted *R*^2^ values are included in the legend. (d) The calculated PFOA surface concentration from eqn (3) as a function of bulk concentration. (e) Integrated C–F peak area of IRRAS measurements of PFOA surface concentration at the air/aqueous interface of ultrapure water and 0.47 M NaCl. All error bars correspond to one standard deviation from the mean.

Three main peaks are present in the C–F vibrational mode region of the IRRAS spectra of PFOA in Fig. 1a (peak assignments are described in the ESI†). The peaks red-shift with increasing bulk PFOA concentration, indicative of increasing adsorption and intermolecular interactions between interfacial PFOA molecules. The aqueous subphase composition has a significant impact on the C–F peak intensities, lineshapes, and frequencies. For example, C–F peaks are not observable for PFOA dissolved in ultrapure water at 2 μM or 20 μM, suggesting that NaCl enhances PFOA adsorption at low concentrations. Peak integration of the C–F vibrational modes (Fig. 1b) further illustrates that NaCl enhances PFOA adsorption at low concentrations. A PFOA concentration of 200 μM brings about an inflection point in this trend wherein the integrated IRRAS peak area of the two aqueous solutions is nearly the same, and at concentrations >200 μM the integrated peak area actually becomes larger in H_2_O than in 0.47 M NaCl.

In response to this apparent deviation from Hofmeister “salting-out” effects,^42,43^ surface tension measurements were collected to further investigate PFOA interfacial adsorption and surface concentration for comparison to the IRRAS spectra. Although surface tension does not provide any molecule-specific information, it does yield robust quantitative measurements of surfactant adsorption to the air/water interface. Enhanced surfactant concentration at the surface, relative to the bulk, results in a lower surface tension than that of H_2_O and corresponds to an increase in surface concentration. The average surface tension of H_2_O was measured at 72.57 ± 0.22 mN m^−1^ at 21.1 ± 0.5 °C, consistent with the reference value.^44^

Fig. 1c illustrates a surface tension depression with increasing bulk PFOA concentration. Surface tension measurements for each aqueous subphase solution were fitted to the Szyszkowski equation,^45,46^2
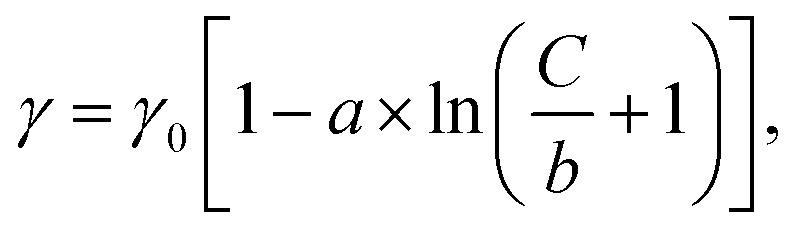
where *γ* is the surface tension of the PFOA solution, *γ*_0_ is the surface tension of the aqueous subphase, *C* is the PFOA concentration in bulk aqueous solution, and *a* and *b* are fitted parameters. The results of the fitting (Fig. 1c) show that the magnitude of surface tension depression induced by interfacial PFOA adsorption is highly dependent upon the presence of salt,^46–50^ as the 0.47 M NaCl solution exhibits decreased surface tension across all bulk PFOA concentrations, relative to H_2_O.

To quantify the differences in PFOA surface concentration induced by the aqueous subphase, the Langmuir–Szyszkowski equation was used to compute the surface concentration, *Γ*:^46,51^3
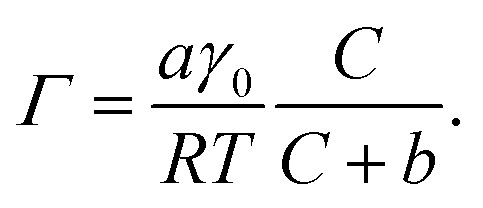


Fitted parameters *a* and *b* from eqn (2) were used in eqn (3) to obtain the calculated surface concentration in Fig. 1d. The aqueous NaCl solution supports an order of magnitude larger PFOA surface concentration than H_2_O, with 10–12× the surface concentration of H_2_O for solutions containing 2 mM PFOA and 2 μM PFOA, respectively. Second harmonic generation (SHG) spectroscopic measurements of the interfacial electric field (Fig. S1†) also confirm enhanced PFOA surface activity in the NaCl solution, consistent with Hofmeister series “salting-out”.

While IRRAS is sensitive to changes in PFOA adsorption due to variable surfactant concentration and aqueous subphase composition, the spectroscopic measurements produce opposite trends relative to the surface tension measurements at concentrations of PFOA exceeding 200 μM. Surface tensiometry indicates that the NaCl subphase enhances PFOA surface adsorption relative to H_2_O across all PFOA concentrations, whereas the IRRAS integrated C–F peak area implies that NaCl provides an initial enhancement of PFOA surface adsorption, followed by an anomalous decline in surface adsorption at PFOA concentrations >200 μM. The same anomaly was observed for IRRAS and surface tension measurements of PFOA interfacial adsorption to a 10 mM CaCl_2_ subphase (ESI Fig. S4†), indicating that the discrepancy cannot be ascribed to electrolyte composition nor concentration alone. Aggregation of PFOA could result in C–F vibrational coupling, explaining the surfactant concentration-dependent discrepancies between the IRRAS and surface tension data. The 0.47 M NaCl subphase also enhances shoulder features in the IRRAS spectra at 1133 cm^−1^ and 1105 cm^−1^, which could be a signature of energy splittings characteristic of vibrational excitons.

In order to highlight deviations from the Beer–Lambert law, we turn to the effective infrared cross section (Fig. 1e). In the absence of vibrational coupling, the integrated peak area would increase linearly with surface concentration, allowing for the extraction of an effective infrared cross section from a linear fit of the data. The integrated peak area of PFOA in water is nearly linear with surface concentration, suggesting nearly ideal “gas-phase” absorption behavior. However, even in neat water there are deviations from ideal absorption at higher concentrations, suggesting that this phenomenon is concentration dependent. This concentration dependence is amplified in the presence of electrolytes (see Fig. S3† for additional data), which induce significant nonlinearity in the C–F peak area as a function of PFOA surface concentration. Concentration dependence could suggest that the deviations from ideal absorption are caused by PFOA⋯PFOA interactions. We investigate the origin of these strong deviations from Beer–Lambert absorption in the context of our exciton hypothesis in what follows.

### Molecular dynamics simulations

We begin the theoretical investigation by obtaining reliable structures of PFOA at the air/water interface using classical MD simulations based on the AMOEBA polarizable force field.^52^ The equilibration timescale for aqueous PFOA is on the order of minutes (as implied by the measured surface tension equilibration time), well beyond the timescales accessible to simulation. In view of this, we allowed simulations of the neat water and NaCl(aq) interfaces to equilibrate for as long as computationally feasible (2.5 ns) to minimize deviations from equilibrium (see Section 4 for details).

**Fig. 2:**
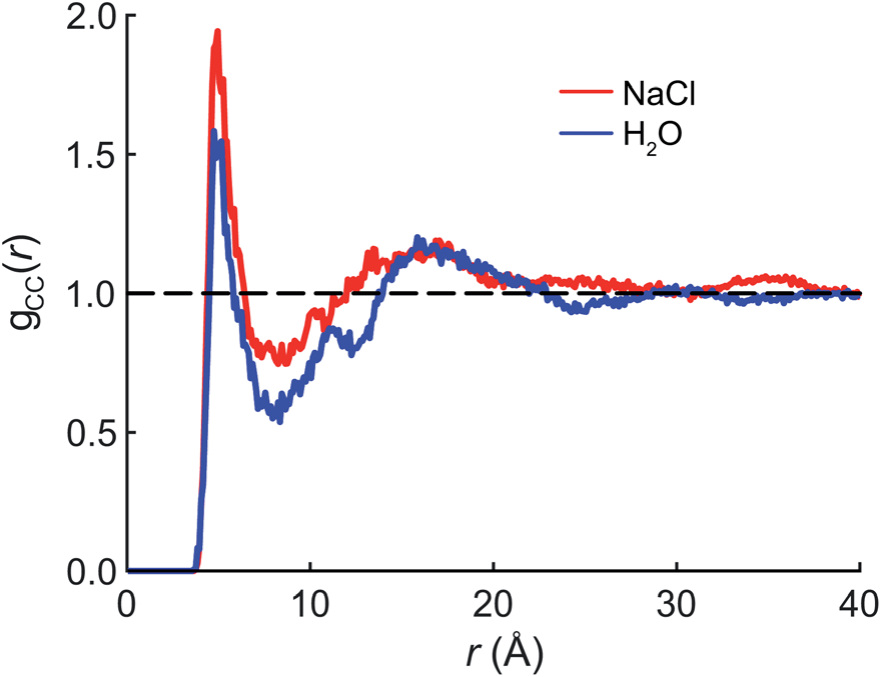
Radial distribution functions for the terminal (CF_3_) carbon atoms in PFOA.

The radial distribution function (RDF) *g*(*r*) between the terminal (CF_3_) carbon atoms in PFOA is shown in Fig. 2. The initial peak at 7.5 Å is larger in the NaCl solution, implying that the PFOA tails are more structured in NaCl solution than in water. The value of *g*(*r*) at the first local minimum is smaller in neat water, implying that the average distance between CF_3_ moieties is larger in neat water despite the fact that the second peak in the RDF occurs at approximately the same value of *r* in both neat water and in NaCl solution. Neither our experiments nor simulations show any evidence of micellization, however a higher propensity for soluble surfactants to form micelles in saline solution is well documented,^53^ supporting the notion that PFOA molecules aggregate more closely in the presence of NaCl. While we are not expressly concerned with micellization, the propensity for molecules to self-aggregate has important consequences for delocalization of the vibrational wavefunctions, as the vibrational couplings decay rapidly (∼*R*^−3^) with distance.

### Vibrational exciton model spectrum

We parameterized a vibrational exciton Hamiltonian following the dipole-coupling model,^4,8,21^ using transition dipole parameters obtained from *ab initio* harmonic vibrational frequencies computed for PFOA at 25 different snapshots spanning 250 ps of the MD simulation. We couple every vibrational mode of all 200 PFOA molecules at each snapshot *via* the *ab initio*-derived transition dipole moments; see the ESI† for details. Noninteracting vibrational modes of each PFOA molecule were computed in vacuum and in the absence of dielectric screening, resulting in a spectrum that reflects the intermolecular coupling of gas-phase PFOA monomers within a solution-phase distribution of positions. Solvent effects are omitted for simplicity, computational efficiency, and to gauge whether they are needed in order to reproduce the experimental spectrum. Vibrational coupling to the solvent is frequently invoked in cases of odd or difficult-to-explain spectral features, and the minimalist model employed here allows us to probe whether coupling to the solvent is actually important in the present case.

**Fig. 3:**
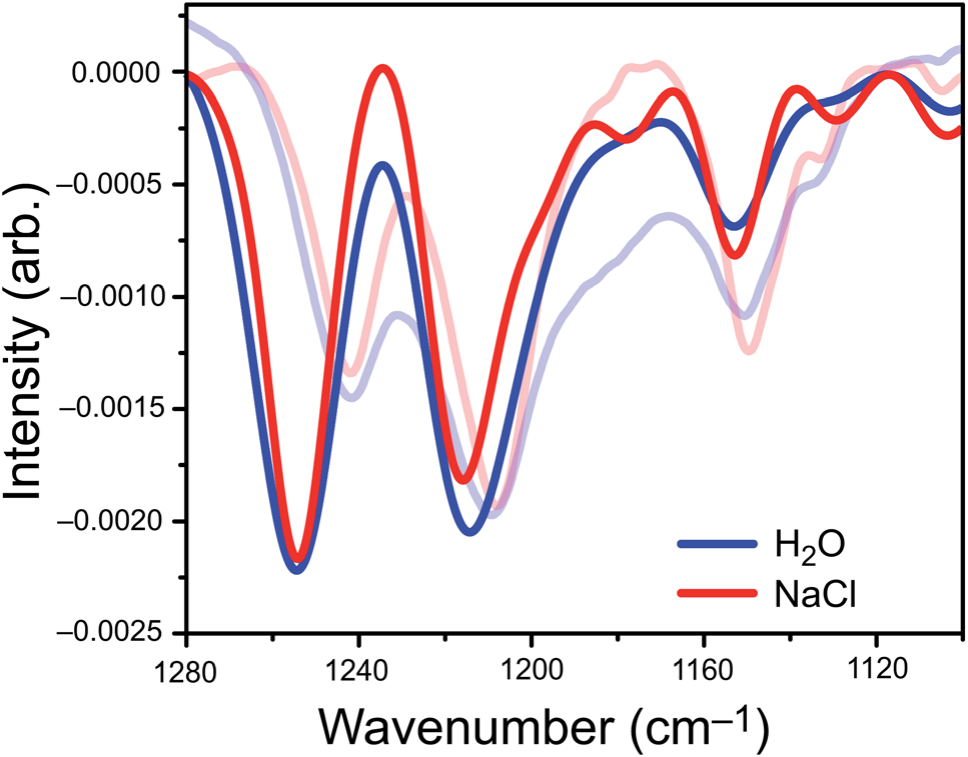
Exciton model spectra (opaque) overlaid with experimental spectra of 1 mM PFOA (translucent). Each band in the exciton spectrum is plotted as a Gaussian lineshape, the width of which is set to the 2*σ* standard deviation in wavenumbers across 25 MD snapshots.

The *ai*VEM spectra in Fig. 3 show exceptionally good agreement with experiment. All center wavelengths are in quantitative agreement (Table 1). Aside from overestimation of the intensity of the highest-energy band, the exciton model spectrum does a remarkable job of reproducing the fine details of the experimental spectrum, including asymmetry of the central band and the appearance of shoulders at 1180, 1130, and 1105 cm^−1^ that are all more pronounced in NaCl than in water. Importantly, the loss of intensity upon introduction of NaCl that is observed in the integrated peak areas is reproduced by the theoretical model.

**Table 1:** Center wavenumbers (in cm^−1^) of major features in IRRAS spectra of PFOA

Subphase	*ω*_1_			*ω*_2_			*ω*_3_		
	*ai*VEM	Expt.	Δ^a^	*ai*VEM	Expt.	Δ^a^	*ai*VEM	Expt.	Δ^a^
H_2_O	1254.0	1241.7	12.3	1214.0	1209.5	4.5	1153.0	1150.5	2.5
NaCl	1254.0	1242.0	12.0	1216.0	1208.0	8.0	1153.0	1149.7	3.3

a Absolute difference with respect to experiment (Δ = |*ω*_*ai*VEM_ − *ω*_expt._|).

Omission of solvent effects in the *ai*VEM allows us to isolate the origins of several important factors that influence the IRRAS spectra of PFOA, because we can examine the role of excitonic coupling in the absence of external perturbations. In particular, we wish to examine the origins of the apparent loss of integrated intensity when salt is added to the aqueous subphase. To do so, we examine the difference spectrum Δ*I* = *I* − *I*_0_ in the experimental window 1100–1280 cm^−1^, where *I* is the intensity obtained from the *ai*VEM and *I*_0_ is the intensity obtained the uncoupled monomers. The uncoupled calculation includes the effects of heterogeneous broadening on the distribution of oscillator frequencies, and therefore the difference Δ*I* is a direct measure of the changes in intensity induced by intermolecular vibrational coupling.

Analysis of Δ*I* (Fig. S6†) reveals that the appearance of small shoulder features in both spectra is characteristic of exciton splitting due to vibrational coupling between C–F oscillators. The small shoulders at 1178 cm^−1^, 1130 cm^−1^, and 1100 cm^−1^ (Fig. 3) are more pronounced in the NaCl spectrum than in neat water, although only the two low-energy shoulders appear to be experimentally resolvable. Near the intensity maxima, vibrational wavefunction delocalization depletes signal intensity due to the expected splitting of the parent peak into *ω*_+_ and *ω*_−_ absorption bands. Overall, vibrational coupling leads a spectrum whose intensity is more diffuse, with oscillator strength distributed across a wider range of frequencies. At least some of the intensity that seems to disappear from the C–F region of the spectrum in salty water is simply pushed outside of the experimental detection window, but the emergence of shoulders and signal depletion of the major bands also contributes to the depletion in observed intensity, as a diffuse set of low-intensity modes may disappear into the background in the experiment. Both effects are directly attributable to vibrational excitons.

**Fig. 4:**
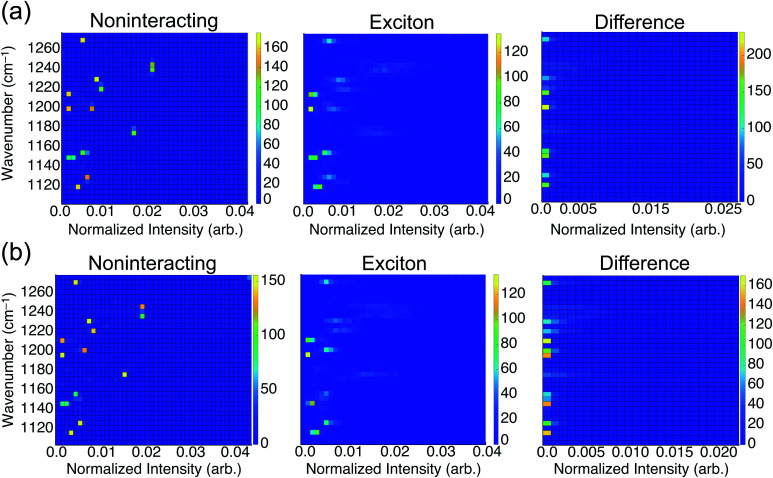
Two-dimensional histograms correlating energy and intensity in (a) neat water and (b) NaCl solution. The “noninteracting” histograms show the distribution of frequencies and intensities obtained from the uncoupled model, 〈*Ĥ*_0_〉, corresponding to intensities *I*_0_ in the text. The “exciton” results correspond to the fully-coupled *ai*VEM (intensities *I*). Differences Δ*I* = *I* − *I*_0_ are shown on the right. All data are averaged over 25 snapshots to incorporate thermal fluctuations.

To consider the overall influence of vibrational coupling on the observed intensities, Fig. 4 presents the distribution of frequencies and intensities obtained from the uncoupled model (intensities *I*_0_ that include thermal broadening effects only) *versus* the fully-coupled *ai*VEM (intensities *I*), along with the difference Δ*I* = *I* − *I*_0_. This analysis reveals the two primary roles played by vibrational coupling in the IRRAS spectra. First, when considering thermal fluctuations but neglecting intermolecular coupling, intensity appears as a set of discrete features at isolated frequencies, whereas the effect of transition dipole coupling is to engender delocalization of the spectral intensity across a wide range of frequencies. This implies that a primary source of band broadening in PFOA is vibrational coupling rather than thermal or heterogeneous broadening.

We assess the net shifts in intensity upon vibrational coupling by correlating excitation energy and the intensity difference between the noninteracting and the fully-coupled spectra. The primary effect of coupling is to generate numerous states with near-zero oscillator strengths, a characteristic intensity-borrowing effect in excitonic spectra.^24^ Comparing the difference spectra by subphase reveals that NaCl solution (Fig. 4b) facilitates delocalization of oscillator strength into a much larger number of low-intensity modes as compared to results in neat water (Fig. 4a). There are also regions in the NaCl correlation plot where small but non-negligible intensity gains appear upon coupling; these features are attributable to the small shoulders present in NaCl solution but absent in neat water. With the intensity distributed over so many modes with near-zero oscillator strengths in NaCl solution, it can be reasonably expected that these contributions may simply vanish into the background noise of an experimental measurement. Each of the new modes that appears by virtue of the coupling borrows only a small amount of intensity from the nominal bright states, but collectively these modes account for a significant fraction of the total oscillator strength. A staggering 54% of vibrational modes for PFOA in NaCl solution have intensities that are an order of magnitude smaller than the most prominent peak in the spectrum.

As mentioned in the Introduction, when integrated across all wavelengths the total oscillator strength must be conserved, but in practice this theoretical limit is not achieved for comparatively diffuse spectra where some of the oscillator strength vanishes into the background. In solids, where these effects are far more pronounced, the influence of vibrational excitons on intensity is almost always reported as signal loss.^3,9–11^ The correlation analysis between frequencies and intensities in the presence or absence of the transition dipole coupling suggests that the reduction in oscillator strength in NaCl solution that is observed experimentally corresponds to precisely this type of signal reduction.

Combining the structural insights from MD simulations, experimental surface tension measurements, and the agreement of the *ai*VEM with experiment, the vibrational exciton hypothesis appears to be the more likely source of the signal reduction as compared to desorption of PFOA back into the aqueous subphase. The exciton hypothesis explains anomalies observed in IRRAS measurements of other soluble surfactants such as sodium dodecyl sulfate, where similar intensity decreases have been noted in the spectra of the C–H tails.^28^ An important question remains as to whether vibrational excitons are unique to soluble surfactants, or if this phenomenon is generalizable to insoluble monolayers. This is addressed below.

The impressive agreement of the *ai*VEM with the PFOA experiments furthermore suggests that this theoretical model constitutes a useful tool with predictive capacity. On the other hand, the C–F bonds in PFOA are somewhat exotic and may be poor surrogates for general trends in self-aggregating lipids at interfaces. As a model of the latter, we therefore applied the *ai*VEM to a 2D sheet of 24 uniformly spaced octanoic acid (C_8_H_16_O_2_) molecules, in an effort to examine the role of vibrational coupling in the 1D spectroscopy of C–H modes in an ordered monolayer at the air/water interface. The results (Fig. S7†) feature a lowering in the local intensity of the C–H vibrational modes upon compression from a diffuse monolayer to one corresponding to 25 Å^2^ per molecule mean molecular area. The prediction that vibrational coupling can induce distance-dependent signal reduction for an insoluble monolayer is next examined experimentally.

### Arachidic acid monolayer measurements

**Fig. 5:**
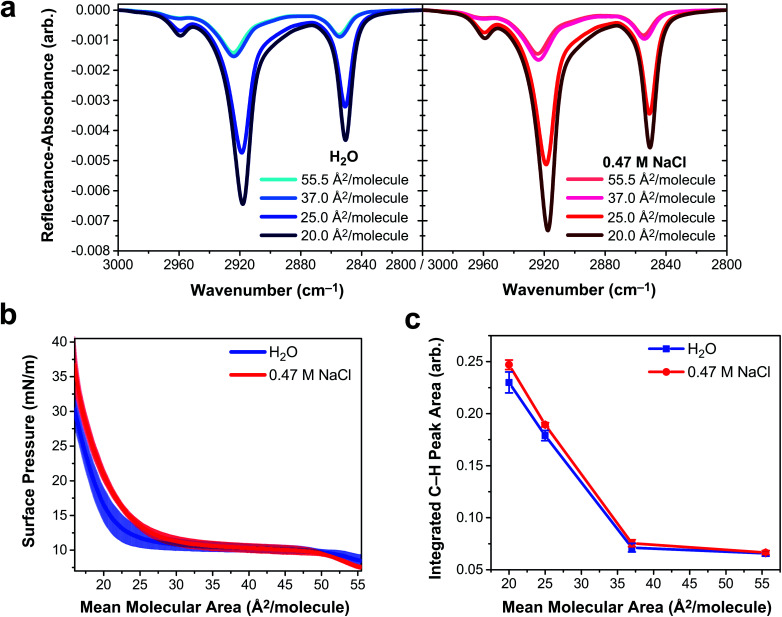
Deprotonated arachidic acid (AA) monolayers on water and 0.47 M NaCl at pH 12.5. Both aqueous subphase solutions contain 4 μM ethylenediaminetetraacetic acid. (a) Infrared reflection–absorption spectroscopy (IRRAS) of the AA monolayer C–H stretching region as a function of mean molecular area, expressed in units of Å^2^ per molecule. (b) Surface pressure–area isotherms of AA monolayers. Shading represents one standard deviation from the mean. (c) Integrated peak area corresponding to C–H vibrational modes as a function of AA monolayer mean molecular area. Lines connecting the data points are drawn to guide the eye, and error bars represent one standard deviation from the mean.

To determine if vibrational excitons can be observed in 1D vibrational spectroscopy of surfactants more generally, an insoluble and deprotonated arachidic acid (AA, C_20_H_39_O_2_) monolayer was probed with IRRAS and surface tensiometry. Deprotonated AA was selected to match the charge state of the deprotonated PFOA surfactants. AA forms a stable monolayer at the air/water interface (Fig. S8†), even when over 95% of the carboxylic acid headgroups are deprotonated at pH ∼ 12,^54^ making it an excellent insoluble surfactant to contrast with PFOA. IRRAS spectra of an AA monolayer on water and on 0.47 M NaCl at pH 12.5 were collected at constant mean molecular area, and a plot of the C–H vibrational modes is shown in Fig. 5a. To prevent trace metal binding to the AA monolayer, 4 μM ethylenediaminetetraacetic acid was added to both subphase solutions.^55^

Mean molecular area values were chosen based on the surface pressure–area isotherms in Fig. 5b. At 55.5 Å^2^ per molecule, the AA monolayer is in a liquid expanded (LE) phase in which the surfactants are fluid and not yet aggregated. Monolayer compression yields a plateau region (37 Å^2^ per molecule) corresponding to a first-order phase transition between the LE and tilted condensed (TC) phases, known as the LE-TC coexistence region.^55,56^ AA surfactants aggregate into 2D domains at the air/water interface and increase in size with compression.^57^ (Brewster angle microscopy images of the AA domain morphologies are shown in Fig. S9†). The monolayer begins to transition to the TC phase starting at 25 Å^2^ per molecule and the transition is complete by 20 Å^2^ per molecule, characterized by a condensed sheet of surfactant. The AA monolayer has a higher surface pressure at mean molecular areas ≲35 Å^2^ per molecule on the NaCl solution subphase, indicating that NaCl expands the monolayer. The chloride counterion is likely to blame for this monolayer expansion, as it has a greater propensity to partition to the air/water interface than sodium, whereupon it intercalates between the carboxylic acid head groups of AA, amplifying electrostatic repulsion.^58,59^

The three peaks in the IRRAS spectra of AA (Fig. 5a) correspond to the *ν*_AS_-CH_3_ stretch at 2959 cm^−1^, the *ν*_AS_-CH_2_ stretch at 2918 cm^−1^, and the *ν*_S_-CH_2_ stretch at 2850.5 cm^−1^.^60^ A mutual feature of these spectra (regardless of subphase identity) is that the region between the two high-energy bands becomes more intense as a function of monolayer compression. This feature is indicative of delocalization of the vibrational wavefunction across a new region in the vibrational manifold, hinting at exciton splitting. At smaller mean molecular area (25.0 and 20.0 Å^2^ per molecule), the C–H peak intensity is greater on 0.47 M NaCl than on H_2_O, as shown in the integrated C–H peak areas in Fig. 5d. As the AA monolayer is compressed to smaller mean molecular area, the C–H peak area of AA on the NaCl solution subphase becomes larger relative to the water subphase. AA surfactants are closer together on the water subphase and therefore subject to stronger vibrational couplings, which decay with distance as *R*^−3^. As a result, excitonic behavior is observable in insoluble surfactant monolayers *via* IRRAS signal intensity depletion in the C–H stretching region.

The observable impact of vibrational excitons on the IRRAS spectra of AA monolayers is smaller in magnitude than that of PFOA, but it is by no means negligible in the AA spectra. A 7% difference in AA C–H peak intensity (Fig. 5c) at 20 Å^2^ per molecule can be attributed to vibrational coupling mediated by the aqueous subphase composition. In the absence of vibrational coupling, the AA spectra on each subphase would be the same. Attempting to quantify this peak intensity difference for PFOA is more complicated as the PFOA molecules are not strictly confined to the interface. In the case of 2 mM PFOA, the difference between neat water and NaCl subphases is 34%. Astoundingly, the Hofmeister “salting out” observed in both surface tension and SHG measurements suggests that this number is only a lower bound to the true difference engendered by vibrational coupling.

### Observable delocalization length

In order to develop a notion of how delocalized the vibrational wavefunction becomes at the air/water interface, we turn to a 2D sheet model of AA. While we report data for a 2D sheet and 3D unit cell models of PFOA, there are several factors that make AA a cleaner model to study. First, the surface presence of AA is well quantified experimentally, and the monolayer shows no evidence of desorption into the aqueous subphase. Second, the effects of dielectric screening between the surfactants can be neglected, as the intermolecular coupling between AA tails is easily assumed to be far removed from the large dielectric of the aqueous medium below. Lastly, the mean molecular area is well known experimentally, and thus the intermolecular separation can be readily transferred to a theoretical model.

**Fig. 6:**
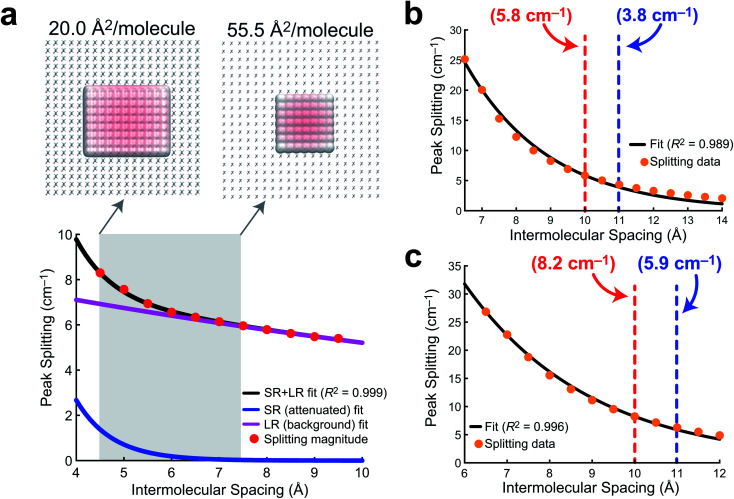
Delocalization length models. (a) A bi-exponential fit of AA exciton (tunneling) splittings for the 2918 cm^−1^ mode as a function of intermolecular spacing (center-of-mass distance) in a model system of 100 AA molecules. The shaded region corresponds to the experimentally probed mean molecular area values. The extent of wavefunction delocalization is visualized at the experimental minimum and maximum mean molecular area values. (b) An exponential fit of exciton splittings of the 1213 cm^−1^ mode for a (10 × 10) 2D PFOA sheet model along a uniform compression coordinate. (c) An exponential fit of exciton splittings of the 1213 cm^−1^ mode for a (5 × 5 × 5) 3D PFOA unit cell with randomly generated PFOA orientations along a uniform compression coordinate. In (b) and (c), the red and blue dashed lines correspond to the nearest neighbor distances from the integrated RDF for NaCl and H_2_O, respectively, and each is annotated with the tunneling splitting at that intermolecular spacing.

In order to quantify the extent of vibrational wavefunction delocalization, Fig. 6 plots the exciton splitting (*ω*_+_–*ω*_−_) for the vibrational mode at 2918 cm^−1^ as a function of intermolecular separation along a uniform expansion coordinate of a 2D sheet model of AA. Fundamentally, the splittings in the exciton model are the quantum mechanical tunneling splittings in multiple-finite-well potentials, which fall off exponentially with distance. Therefore, we fit a bi-exponential function *A*exp(−*αr*) + *B*exp(−*βr*) where *A* and *B* are normalization constants and *α* and *β* are related to the tunneling length scales at short range and long range, respectively. In the short range, the Coulomb potential between classical dipoles diverges, hence the long-range portion of the fit represents classical dipole physics.

The result of the bi-exponential fit (*A* = 536.7, *B* = 8.739, *α* = 1.326 Å^−1^, and *β* = 0.052 Å^−1^) is shown in Fig. 6a. The long-range exponential function determines the delocalization length, and integration suggests that 90% of the wavefunction is contained in the relatively large delocalization length of 23 Å (which is still an order of magnitude smaller than delocalization length scales reported in solids).^3^ Despite the sizable delocalization length, the differences in the splittings as a function of mean molecular area do not become particularly pronounced until after the monolayer is compressed into the region governed by the short-range exponential, ∼6 Å or 36 Å^2^ per molecule. Interestingly, this is precisely consistent with experiment, where the differences in integrated C–H peak intensities are not pronounced until a compression of 25 Å^2^ per molecule. Experimentally, we have shown that the intermolecular spacing on the 0.47 M NaCl subphase is more diffuse than on neat water, and the influence of vibrational wavefunction delocalization is exponentially more pronounced as a function of the intermolecular spacing in the short range. Our model shows that this exponential is governed by a rather large coefficient, suggesting that these subtle changes in the intermolecular packing between the two subphases will cause large enough differences in the measured intensities to be noticeable in 1D IRRAS spectra.

The PFOA models were studied at larger intermolecular spacings, and therefore required only a simple exponential fit (*A*exp[−*αr*]), as the dipole–dipole potential is not divergent at long range. The results of these fits for a 2D sheet model (Fig. 6b) and a 3D unit cell in which the orientations of the PFOA molecules were generated *via* the application of random rotations (Fig. 6c) are consistent with one another despite differences in orientation. The normalization parameters are *A* = 354.3 for the 2D sheet and *A* = 240.8 for the 3D cell. The differences in normalization are expected, as the systems have different dimensionality and contain different numbers of molecules. The interesting number is the tunneling coefficient, where *α* = 0.41 Å^−1^ for the 2D sheet and *α* = 0.34 Å^−1^ for the 3D unit cell. The tunneling coefficients (and the splitting magnitudes themselves) are very similar between models, suggesting that the major difference between the two is simply a shift to longer delocalization lengths due to the stronger interactions in the 3D unit cell. As expected, the additional degree of freedom of translational motion amplifies interactions between transition dipole moments simply by nature of there being more nearby interactions per PFOA monomer. In comparison with the AA data, the exciton splittings in PFOA are larger due to the significantly larger transition dipole moment of the PFOA transition (0.5 D) relative to the AA transition (0.25 D). However, due to transition dipole moment alignment in the more ordered 2D sheet motif, along with less intermodal transition dipole cancellation due to the more ordered molecular geometry of AA (staggered rather than helical), the AA delocalization length is much larger, as *β* = 0.05 Å^−1^ ≪ *α* = 0.4 Å^−1^. Therefore the exciton splitting in PFOA is larger within the space where PFOA molecules interact, and the interactions are supplemented by the partial solubility of PFOA, but they are more short-ranged than in their hydrocarbon counterparts.

In terms of the measurable impact on the 1D IRRAS spectra, our models corroborate the experimental data quite nicely. Although the delocalization is far more significant in AA, the subtle differences in packing structure at the interface due to the composition of the subphase will only alter the vibrational exciton splittings by fractions of a wavenumber per vibrational mode. The result is an effect that is noticeable, but subtle. As for the PFOA spectra, the tunneling splittings that result from differences in subphase composition are augmented by the 3D nature of the PFOA interactions and yield far more significant multiple wavenumber splittings, leading to a more pronounced decline in the local intensity. The combination of theory and experiment presented here strongly suggests that vibrational wavefunction delocalization is to blame for the perceived signal losses in 1D IRRAS of surfactants at the air/water interface.

## 3 Conclusions

We observed the signature of vibrational excitons in surfactants at the air/water interface *via* alkyl and fluoroalkyl vibrational mode signal reduction in 1D surface-sensitive infrared reflection–absorption spectroscopy (IRRAS). Surface tensiometry, molecular dynamics simulations, and *ab initio* calculations confirm the existence of significant vibrational coupling in both soluble fluoroalkyl and insoluble alkyl surfactants. Intentionally excluding solvent effects from the theoretical model reveals that coupling with the solvent is unimportant in obtaining the most pertinent features of the IRRAS spectrum. Instead, model IRRAS spectra of perfluorooctanoic acid obtained from molecular dynamics simulations and vibrational exciton calculations agree nearly quantitatively with experiment, despite exclusion of solvent effects, indicating that surfactant intermolecular coupling dominates the observed spectral features. While solvent effects are minimal, the ionic composition of the aqueous phase plays a significant role in modulating vibrational coupling *via* the strong *R*^−3^ distance dependence of transition–dipole interactions. Salts modulate intermolecular distances (and therefore vibrational couplings) between surfactants, depleting infrared intensity of 1D surface-sensitive spectra. In light of this new interpretation, 1D vibrational spectroscopic studies of surfactants at surfaces of varying aqueous phase compositions should be carefully scrutinized.

The results described above present both challenges and opportunities for the surface science community. Quantitative analyses involving vibrational mode peak intensities must be approached with caution to avoid misinterpretation of vibrational spectra due to vibrational coupling. The molecular environment has a profound impact on the organization of self-aggregating molecules, making fluid interfaces particularly susceptible to the influence of vibrational excitons. On the other hand, the emergence of vibrational excitons in 1D spectra offers a new, highly-sensitive spectroscopic handle for probing molecular organization at interfaces. Further study of the spectral perturbations induced by vibrational excitons is needed for the development of improved 1D surface-sensitive vibrational spectroscopic analyses. Borrowing from the 2D-IR community, future work should focus on leveraging isotope effects (such as dilute mixtures of deuterated AA or ^13^C-labeled PFOA) to purge quasi-degeneracies from the vibrational manifold, allowing for 1D analyses that are free of the impact of exciton delocalization.^61–65^ More broadly, the influence of vibrational excitons should be investigated in systems beyond the air/water interface, particularly in confined environments like aerosols.

## 4 Methods

### Chemicals

Pentadecafluorooctanoic acid (PFOA, 96%, ACROS Organics™) and arachidic acid (AA, ≥99%, Sigma-Aldrich) were used as received. All glassware was cleaned in a piranha acid bath. Solutions were prepared in ultrapure water with a resistivity of 18.2 MΩ cm (Milli-Q Advantage A10, EMD Millipore), and NaCl (sodium chloride, 99.5%, for biochemistry, ACROS Organics™) was baked in a furnace at 650 °C for at least 10 h to remove residual organic impurities.^66^ PFOA was dissolved in ultrapure water and in an aqueous 0.47 M NaCl solution, and the pH of all PFOA solutions was measured at 5.8 ± 0.1 due to acidification by atmospheric CO_2_. AA was dissolved in chloroform (Reagent ACS, 99.8+%, ACROS Organics™) at 1.0 mM. The ultrapure water and 0.47 M NaCl aqueous solution subphases for AA measurements contained 4 μM ethylenediaminetetraacetic acid (EDTA, 99.995% trace metals basis, Sigma-Aldrich) and were pH adjusted with sodium hydroxide pellets (NaOH, 98%, extra pure, ACROS Organics™) to pH 12.5 ± 0.1.

### Surface tensiometry

PFOA surface tension measurements were collected in borosilicate glass Petri dishes using a force tensiometer (Sigma 703D, Biolin Scientific) and the Wilhelmy plate method. A platinum Wilhelmy plate was cleaned with ethanol and ultrapure water and fired until red hot with a Bunsen burner. PFOA solution surfaces were allowed to equilibrate for 3 minutes prior to recording the surface tension, and all measurements were repeated at least five times. Curve fitting of eqn (2) was performed using the Nonlinear Curve Fit Tool in OriginPro 9.0 (OriginLab 9).

Surface pressure–area isotherms of AA were conducted in triplicate using a Teflon Langmuir trough and Delrin barriers (Biolin Scientific), and surface tension was measured with a platinum Wilhelmy plate. Surface cleanliness, indicated by a surface pressure value ≤0.2 mN m^−1^, was assessed by sweeping the barriers across the subphase at the maximum compression speed (270 mm per min per barrier). AA was spread dropwise onto the aqueous subphase, and the chloroform solvent was allowed to evaporate for 10 minutes. The barriers were symmetrically compressed at a rate of 5 mm per min per barrier during the isotherm, and the data were averaged.

### Infrared reflection–absorption spectroscopy

Infrared–reflection absorption spectroscopy (IRRAS) was conducted using a Fourier transform infrared spectrometer (Spectrum 100, PerkinElmer) equipped with a liquid nitrogen-cooled HgCdTe (MCT) detector. Spectra were collected with unpolarized light in the single-beam mode as an average of 400 scans. The incident beam direction inside the spectrometer was modified by a planar gold mirror at a 48° angle of incidence relative to surface normal, and the reflected light was redirected toward the detector with a second gold mirror. Energy values were recorded every 0.5 cm^−1^ between 450 and 4000 cm^−1^, and the spectral resolution was 4 cm^−1^. Each experiment was repeated in at least triplicate and analyzed using OriginPro 9.0. For analysis of the PFOA C–F vibrational modes, the baseline was subtracted from each spectrum by fitting a line between endpoints 1118 and 1266.5 cm^−1^, and the peak area of each baseline-subtracted spectrum was integrated between these endpoints. The C–H spectral region of AA was baseline-subtracted by fitting a line between endpoints 2800 and 3000 cm^−1^, and the peak area was integrated between the same endpoints. Figures contain averaged, baseline-subtracted spectra.

### Molecular dynamics

AMOEBA parameters for anionic PFOA were obtained using a standard protocol as described in the ESI.† Simulations were carried out in the canonical ensemble using slab boundary conditions, with a simulation cell size of 9.5 × 9.5 × 29.5 nm^3^. The temperature was maintained every 0.1 ps at 298 K using a Bussi thermostat,^67^ with a step size of 2 fs maintained *via* the RESPA integrator.^68^ All simulations were run using a locally-modified version of Tinker-HP, v. 1.1.^69^ Our neat water simulations contain a 1 : 1 ratio of Na^+^ : PFOA in order to maintain charge neutrality for application of periodic boundary conditions *via* Ewald summation. NaCl simulations use a 3 : 1 ratio of Na^+^ : PFOA and 2 : 1 Cl^−^ : PFOA ratio to achieve charge balance, for a net 2 : 1 concentration ratio of NaCl : PFOA. The net concentration of PFOA was set to 200 molecules in a 9.5 nm^3^ box, with enough water to provide a density of ≈1 g cm^−3^.

### Quantum chemistry calculations

Vibrational spectra of PFOA were obtained using a vibrational exciton model as described in the ESI.† The exciton model requires normal mode frequencies and transition dipole moments for each monomer. These are computed at the EDF2/6-31G(d) level^70^ (using Q-Chem v. 5.3),^71^ and scaled by a factor of 0.9805.^72^

## Author contributions

K. A. C.-F. and K. C.-F. designed the study, and H. C. A. and J. M. H. supervised the project. K. A. C.-F. and M. E. F. performed the experiments and analyzed the data. K. A. C.-F. designed the simulation protocol and K. C.-F. conducted the molecular dynamics simulations. K. A. C.-F. conceived the vibrational exciton hypothesis, and K. C.-F. mathematized the concept *via* the *ab initio* vibrational exciton model and performed the associated quantum chemistry calculations. K. A. C.-F., K. C.-F., and M. E. F. wrote the manuscript with input from all authors.

## Conflicts of interest

J. M. H. serves on the board of directors of Q-Chem Inc.

## Supplementary Material

SC-012-D1SC01276B-s001
