# Supplementary material for: Vibrational exciton delocalization precludes the use of infrared intensities as proxies for surfactant accumulation on aqueous surfaces
Source: Chem Sci. 2021 May 18;12(24):8320–32. doi: 10.1039/d1sc01276b (PMC8221057; doi:10.1039/d1sc01276b)
Supplement: SC-012-D1SC01276B-s001 [file SC-012-D1SC01276B-s001.pdf]

Electronic Supplementary Material (ESI) for Chemical Science.
This journal is © The Royal Society of Chemistry 2021

Electronic Supplementary Information for: "Vibrational exciton delocalization precludes the use of infrared intensities as proxies for surfactant accumulation on aqueous surfaces"

Kimberly A. Carter-Fenk, Kevin Carter-Fenk, Michelle E. Fiamingo,*
Heather C. Allen,† and John M. Herbert‡
Department of Chemistry & Biochemistry, The Ohio State University, Columbus, OH

April 29, 2021

Contents

S1 Perfluorooctanoic Acid C–F Peak Assignments S2

S2 Langmuir-Szyszkowski Equation S2

S3 Second Harmonic Measurements of PFOA Adsorption S3

S4 PFOA Adsorption at an Aqueous $CaCl_2$ Surface S4

S5 AMOEBA Force Field Parameterization S7

S6 Vibrational Exciton Model S8

S7 Arachidic Acid Monolayer Stability S10

References S13

*Current address: Dept. of Chemistry, University of North Carolina, Chapel Hill, NC
†allen@chemistry.ohio-state.edu
‡herbert@chemistry.ohio-state.edu

S1 Perfluorooctanoic Acid C–F Peak Assignments

Vibrational mode peak assignments of deprotonated perfluorooctanoic acid (perfluorooctanoate, PFO) were performed via geometry optimization and harmonic vibrational frequency analysis using Q-Chem v. 5.3.1.[1] Density functional theory (DFT) at the EDF2/6-31G(d) level of theory was used,[2] and a scaling factor of 0.9805 was applied to the harmonic frequencies.[3] Vibrational frequencies, intensities, and peak assignments corresponding to the C–F vibrational modes are tabulated in Table S1.

Table S1: Peak Assignments Corresponding to the C–F Vibrational Modes of PFO

**Wavenumber (cm^{-1})**	**Intensity (km/mol)**	**Vibrational Mode Assignments**
1095.72	36.734	CF_2 and CF_3 Wagging
1104.99	70.264	CF_2 Asymmetric Stretching
1125.64	23.161	CF_2 Symmetric Stretching and Wagging
1130.98	61.766	CF_2 Symmetric Stretching and Wagging, CF_3 Wagging
1153.29	194.923	CF_2 Symmetric and Asymmetric Stretching
1173.79	15.306	CF_2 Asymmetric Stretching
1177.81	77.910	CF_2 Asymmetric Stretching
1188.97	27.910	CF_2 and CF_3 Asymmetric Stretching
1197.64	93.936	CF_2 Asymmetric Stretching
1206.15	94.093	CF_3 Asymmetric Stretching and C–C Stretching
1214.10	245.849	CF_2 Asymmetric Stretching<br>CF_3 Asymmetric Stretching<br>C–C Stretching
1219.89	243.765	CF_2 and CF_3 Asymmetric Stretching
1245.74	59.383	CF_3 Asymmetric Stretching and C–C Stretching
1254.89	533.145	CF_3 Asymmetric Stretching
1271.12	36.907	C–C Stretching, CF_3 Wagging
1284.95	40.870	C–C Stretching, CF_3 Wagging
1310.55	25.624	C–C Stretching, CF_3 Wagging

S2 Langmuir-Szyszkowski Equation

To quantify the differences in PFOA interfacial concentration induced by the ions dissolved in aqueous solution, a modified form of the Gibbs adsorption isotherm was used.[4] The first derivative of the Szyszkowski equation [Eq. (2)],

$$\gamma_0 \frac{d}{dC}\left[1 - a \times \ln\left(\frac{C}{b}\right)\right] = -\frac{a\gamma_0}{C+b}\ , \tag{S1}$$

was inserted into the Gibbs equation,[5]

$$\Gamma_{\text{ex}} = -\frac{C}{RT}\left(\frac{\partial \gamma}{\partial C}\right)_T\ , \tag{S2}$$

to produce the Langmuir-Szyszkowski equation:[6;7]

$$\Gamma = \frac{a\gamma_0}{RT}\frac{C}{C+b}. \tag{S3}$$

In Eqs. (S1)–(S3), γ_0 is the surface tension of the aqueous subphase solution, γ is the surface tension of the PFOA solution, C is the PFOA concentration in bulk aqueous solution, a and b are fitted parameters, Γ_{ex} is the PFOA surface excess, Γ is the surface concentration, R is the gas constant, and T is the temperature.

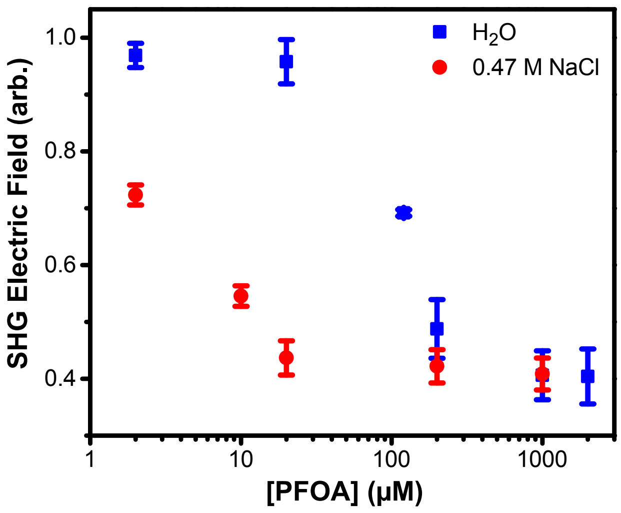

Figure S1: Second harmonic generation (SHG) spectroscopic measurements of perfluorooctanoic acid (PFOA) adsorption at the air-aqueous interface of ultrapure water and 0.47 M NaCl solution. Error bars represent one standard deviation from the mean.

S3 Second Harmonic Measurements of PFOA Adsorption

Second harmonic generation (SHG) spectroscopic measurements of the interfacial electric field as a function of bulk aqueous composition and PFOA concentration were conducted to confirm the surface tension measurements of PFOA interfacial adsorption. A custom-built SHG spectrometer was used for these measurements, and the instrument design has been described previously.[8] Sub-50 fs pulses centered at 805 nm were generated using an ultrafast oscillator (Tsunami, Spectra-Physics) with an output power of ∼900 mW and a repetition rate of 82 mHz. A beam splitter directed 90% of the laser output power into a signal channel, and the remaining 10% was directed to a reference channel where a BBO crystal (90015087, Newport) generated the reference SHG signal. The signal channel consisted of a Glan-laser polarizer (10GR08-AR16, Newport), a half-wave plate (10RP52, Newport), and a long-pass filter (690LP RapidEdge, Omega Optical). Linear p-polarization was selected for the incoming beam polarization to maximize the measured signal intensity. The laser pulses were focused onto the aqueous surface, and the reflected SHG photons were collimated through a focusing lens. The collimated signal then passed through two short-pass filters (450SP RapidEdge, Omega Optical), a half-wave plate (WPH10M-405, Thorlabs), and a Glan-laser polarizer (GLB10-405, Thorlabs). Linear p-polarization was also selected for the SHG signal polarization for maximum signal intensity. A silica plate aligned the reference beam parallel with the signal beam, and both beams were focused through a monochromator slit (Shamrock 303i, Andor) and onto an EMCCD camera (Newton DU970N-BV EMCCD, Andor). Second harmonic images were collected with an exposure time of 90 s and 200× electron multiplication. The CCD background intensity was subtracted from the SHG signal and reference intensities, and the SHG sample signal was normalized to the reference. The normalized SHG signal intensities were then converted to electric field values by taking the square root of the intensity values. The SHG electric field data points plotted in Fig. S1 are the averages of triplicate measurements, and the error bars represent one standard deviation from the mean.

Unlike hydrogenated alkyl surfactants, perfluorinated alkyl surfactants decrease the interfacial electric field relative to that of water.[9;10] Thus, as the PFOA interfacial concentration increases, the SHG electric field decreases. Aqueous NaCl enhances the surface activity of PFOA, leading to SHG electric field and surface tension depression as a function of PFOA bulk concentration. The interfacial electric field of PFOA in ultrapure water decreases minimally at 2 and 20 μM, matching the minimal decrease in surface tension

shown in Fig. 1c. PFOA adsorption at the surface of both ultrapure water and 0.47 M NaCl aqueous solution converge upon a minimum SHG electric field value near 0.4 AU, suggesting PFOA saturation within the SHG probe region. However, the SHG signal also nears the limit of detection within this high PFOA concentration regime, making the full adsorption isotherm difficult to measure. Surface tension and infrared reflection-absorption spectroscopy (IRRAS) have deeper probe depths than SHG, so it is plausible that the SHG measurements are only sensitive to the first interfacial monolayer of PFOA. IRRAS interfacial probe depth corresponds to the IR penetration depth in water at the wavelengths of interest, so the IR penetration depth is $\sim 19\ \mu$m in the C–F vibrational mode region.[11] Consequently, the IRRAS probe depth more closely matches that of the surface tension measurements than the SHG measurements, and the surface tension and IRRAS measurements likely detect multiple layers of PFOA interfacial adsorption.

S4 PFOA Adsorption at an Aqueous $CaCl_2$ Surface

PFOA adsorption to a 10 mM $CaCl_2$ aqueous surface was measured with IRRAS and surface tension. The PFOA C–F modes are plotted in Fig. S2a, and the integrated peak area (Fig. S2b) is compared with that of PFOA in ultrapure water. As PFOA bulk concentration increases from 2 μM to 2000 μM, the C–F peak intensities increase. Additionally, the C–F peaks broaden and red-shift with increasing concentration, indicative of increased intermolecular interactions between interfacial PFOA molecules. Similar to the spectroscopic observations of PFOA adsorption to the 0.47 M NaCl aqueous solution interface, the Ca^{2+} cations salt-out the PFOA surfactants and increase PFOA surface activity at low concentrations, as shown by the greater integrated C–F peak areas for the aqueous $CaCl_2$ subphase at [PFOA] $< 1000\ \mu$M. At 1000 μM PFOA, the water and 10 mM $CaCl_2$ subphase integrated peak areas are approximately the same, and the integrated peak area of PFOA in water is greater than that of the aqueous $CaCl_2$ subphase at 2000 μM PFOA. The same trend reversal in the integrated C–F peak areas was observed between the water and 0.47 M NaCl (aq) subphases at PFOA concentrations exceeding 200 μM (Fig. 1b), indicating that the phenomenon is not specific to a particular salt or concentration.

Surface tension measurements were conducted to further investigate the anomalous IRRAS measurements. Lowered surface tension values of the PFOA solutions relative to that of the aqueous subphase alone indicate surfactant interfacial adsorption. The surface tension of PFOA in 10 mM $CaCl_2$ aqueous solution was lower across all PFOA concentrations (2–2000 μM) relative to that of PFOA in water (Fig. S2c), indicating enhanced surface activity in the presence of salt. To better quantify PFOA adsorption, surface concentration was calculated from the Langmuir-Szyszkowski equation; the surface tension measurements of each aqueous subphase were fit to the Szyszkowski equation [Eq. (2)], and the fitted parameters were used in Eq. (S3) to obtain PFOA surface concentration (Fig. S2d). PFOA surface concentration in 10 mM $CaCl_2$ was approximately 5× greater than PFOA surface concentration in H_2O. The PFOA surface concentration in 0.47 M NaCl was approximately twice as large as the 10 mM $CaCl_2$ subphase surface concentration, indicating that the Ca^{2+} ion has a significant effect on PFOA partitioning at the air-water interface despite the ionic strength of the NaCl solution subphase being over 15× greater than the $CaCl_2$ solution subphase ionic strength.

The surface tension measurements of PFOA in 10 mM $CaCl_2$ are in agreement with the IRRAS measurements at PFOA concentrations less than 1000 μM; however, the trends diverge at PFOA concentrations greater than 1000 μM. This discrepancy in the surface tension and IRRAS measurements at high PFOA concentrations was also observed between the 0.47 M NaCl and water subphases. Thus, the disagreement between surface tension and IRRAS measurements cannot be attributed solely to the ionic composition, concentration, or ionic strength of the aqueous solution. Instead, the ions impact the intermolecular spacings between PFOA molecules, thereby modulating the extent of vibrational coupling between surfactants and the magnitude of IRRAS signal reduction in the C–F vibrational mode region.

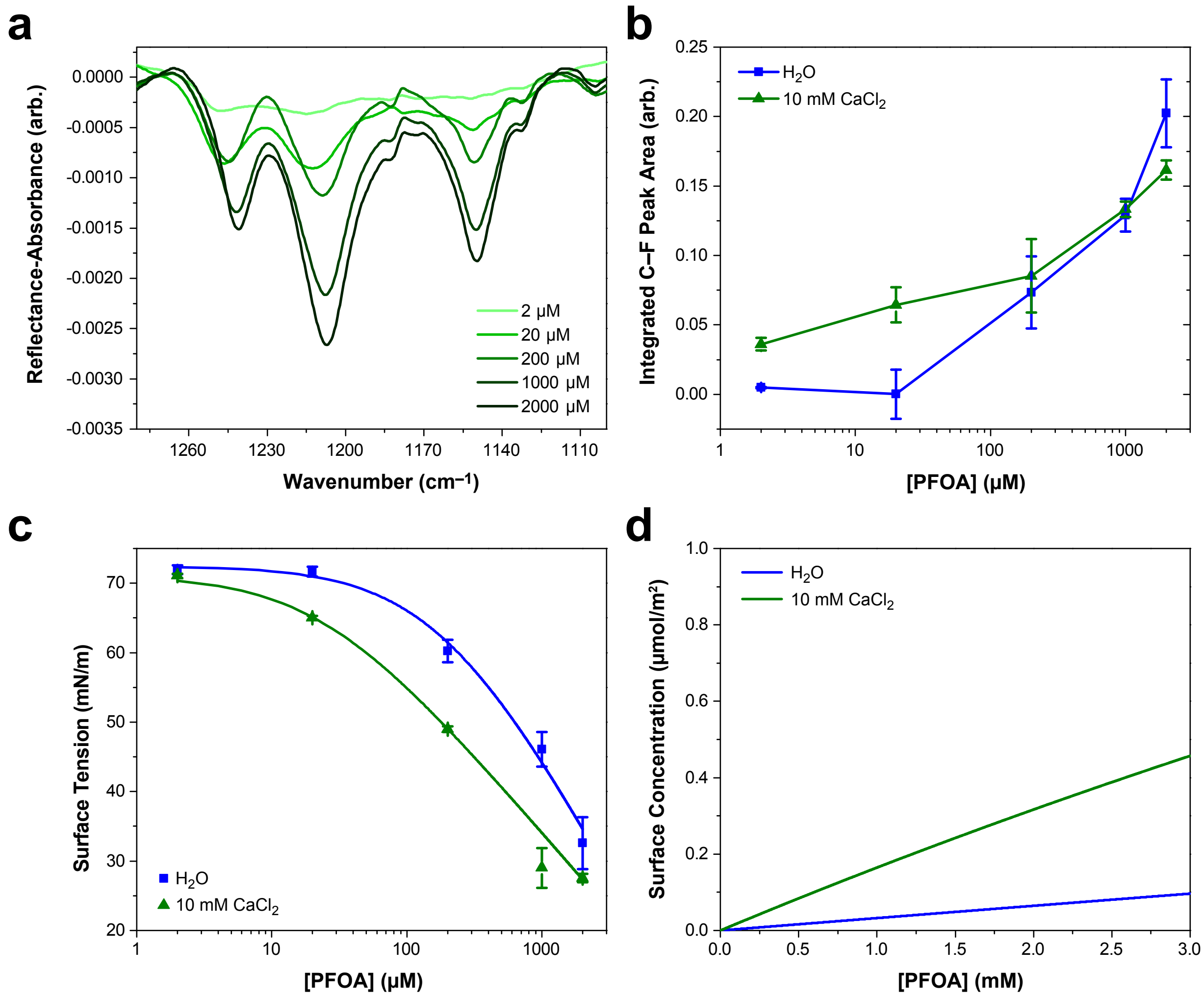

Figure S2: Perfluorooctanoic acid (PFOA) adsorption to the ultrapure water and 10 mM $CaCl_2$ aqueous solution interface. (**a**) Infrared reflection-absorption spectroscopy (IRRAS) of the C–F vibrational mode region as a function of PFOA bulk concentration. (**b**) Integrated peak area corresponding to the C–F vibrational modes as a function of the PFOA bulk concentration. Lines connecting the data points are drawn to guide the eye. (**c**) Surface tension measurements as a function of PFOA bulk concentration. The curved lines connecting the data points are fits to the Szyszkowski equation [Eq. (2)]. (**d**) Calculated PFOA surface concentration from Eq. (S3) as a function of bulk concentration. All error bars correspond to one standard deviation from the mean.

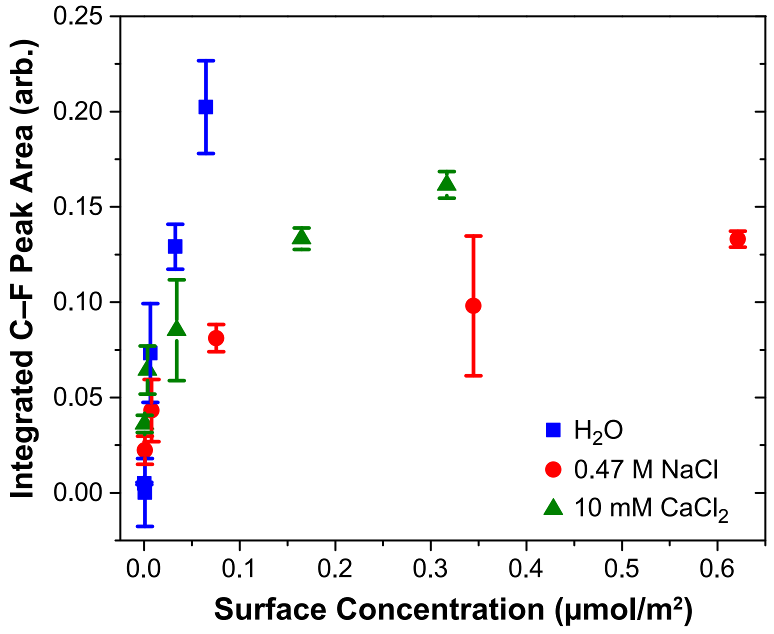

Figure S3: Integrated C–F peak area of infrared reflection-absorption spectroscopic (IRRAS) measurements of perfluorooctanoic acid (PFOA) surface concentration at the air-aqueous interface of ultrapure water, 0.47 M NaCl, and 10 mM $CaCl_2$. Error bars represent one standard deviation from the mean.

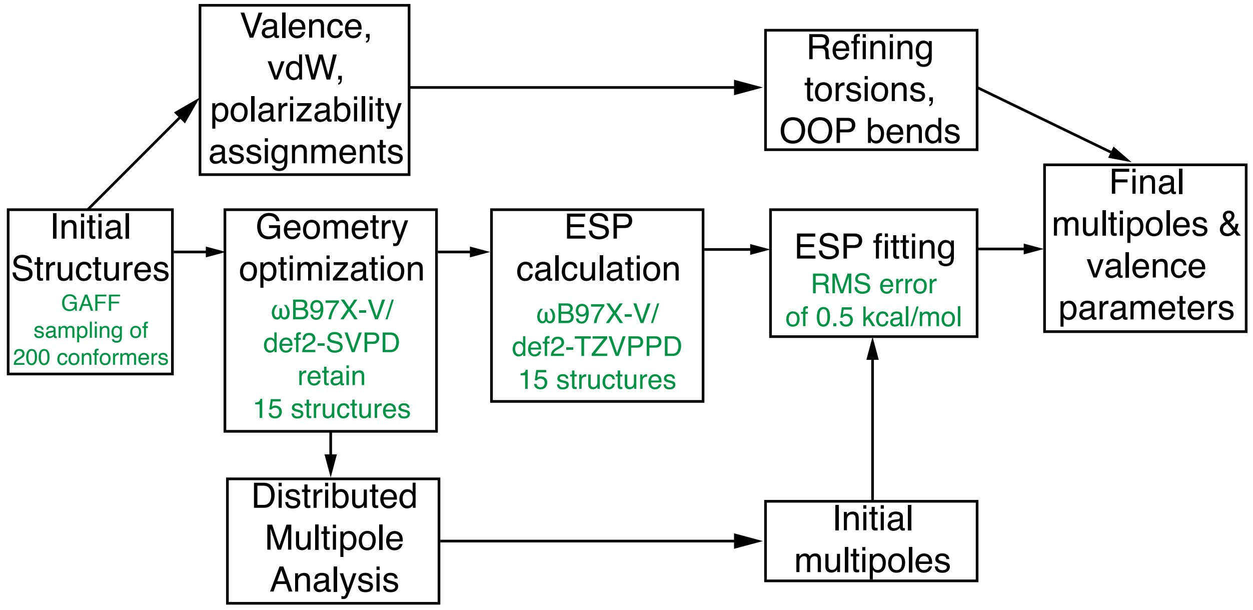

Figure S4: Protocol for parameterizing anionic PFO for use with the AMOEBA force field. Adapted from the flowchart in Ref. 12

S5 AMOEBA Force Field Parameterization

Molecular dynamics simulations were carried out using the AMOEBA force field but parameters for PFO were unavailable and we obtained via the following standard procedure.[13] The initial atomic multipoles were obtained from Anthony Stone's Generalized Distributed Multipole Analysis (GDMA) program,[14] interfaced with the Psi4 quantum chemistry package.[15] We used the original implementation, where all atomic radii are set to 0.65 Å, and the calculation of the distributed multipoles is consistent with the earlier DMA procedure.[16] The initial multipoles were derived from the electron density of a linear (helical) PFO molecule, optimized at the ωB97X-V/def2-TZVPPD level. These multipole moments were fitted to the electrostatic potential of 15 configurations of PFO using the `Potential` package in the Tinker software,[17] in order to more accurately represent the electrostatic potential across multiple conformers. The conformers were obtained from an initial sampling of 200 structures obtained with the generalized AMBER force field (GAFF),[18] which were later optimized at the ωB97X-V/def2-SVPD level. The 15 lowest-energy conformers were chosen as representative of the structural flexibility of PFOA, and *ab initio* electrostatic potentials were computed at the ωB97X-V/def2-TZVPPD level using Psi4. Atomic multipoles were fitted to the electrostatic potential of the 15 low-energy conformers until a root-mean-squared (RMS) error of 0.5 kcal/mol was reached.

Finally, valence parameters were taken from Tinker's `Valence` program. The out-of-plane bend parameters for the carboxylate head group afforded the largest errors when compared with the *ab initio* geometry, and were refined by minimizing the RMS error against the *ab initio* geometry. The final parameters give excellent agreement with the gas-phase geometry of PFOA, capturing the helical structure of the CF_2 moieties that we find to be absent in almost all other force fields (including UFF, MMFF94, MMFF94s, and Ghemical). The overall procedure along with the levels of theory used at each step is outlined in the flowchart in Fig. S4.

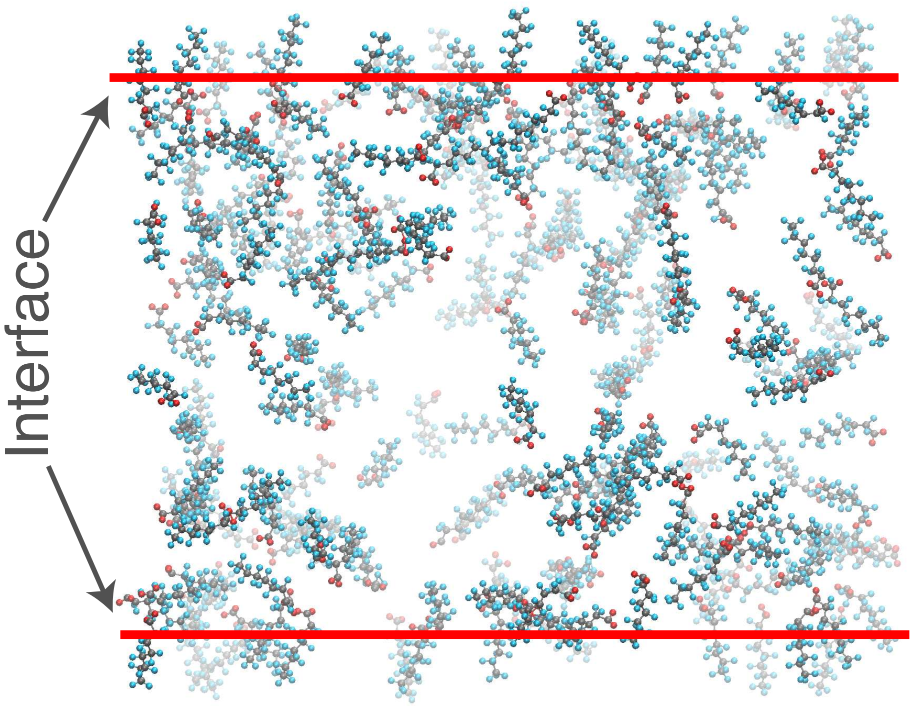

Figure S5: Molecular dynamics snapshot of PFOA where all water has been rendered invisible for ease of inspection. The air/water interface has been labelled to aid in interpretation.

The results of our parameterization are showcased in the molecular dynamics snapshot in Fig. S5, where the PFOA molecules appear to behave in accordance with expectations. As a semi-soluble surfactant, PFOA has a sizable presence in the bulk region between the two interfaces, but shows a strong affinity for the air/water interface. The PFOA molecules also sample far more conformational motifs in bulk solvent than at the interface where their structures are consistently rigid and helical. These rigid helical structures are reminiscent of the gas-phase PFOA geometry, which is sensible as the hydrophobic tails are vacuum exposed.

S6 Vibrational Exciton Model

Vibrational exciton models couple the *resonant* dipole moments between sites. The vibrational coupling between individual monomers is calculated using information from *ab initio* normal mode analysis on individual sites. These sites (or fragments) are taken to be individual surfactant molecules.

The vibrational exciton Hamiltonian takes the form

$$\hat{H} = \sum_{A=1}^{N} H_{0,A} + \sum_{A<B}^{N} \sum_{i \in A}^{M_A} \sum_{j \in B}^{M_B} \boldsymbol{\mu}_i \mathbf{T}_{ij} \boldsymbol{\mu}_j \ , \tag{S4}$$

where $\hat{H}_{0,A}$ is the vibrational Hamiltonian of isolated molecule A and $\boldsymbol{\mu}_i$ is the transition dipole moment of normal mode i. Coupling between the resonant transition dipole moments takes the usual form of the dipole-dipole interaction tensor $\mathbf{T}_{ij}$, namely

$$4\pi\epsilon_0 \mathbf{T}_{ij} = \hat{\nabla}_i \otimes \hat{\nabla}_j \left(\frac{1}{R}\right) . \tag{S5}$$

The Cartesian elements $T_{ij}^{\alpha\beta}$ of this tensor ($\alpha, \beta \in \{x, y, z\}$) are

$$4\pi\epsilon_0 T_{ij}^{\alpha\beta} = \frac{3R_{ij}^{\alpha} R_{ij}^{\beta} - R_{ij}^2 \delta_{\alpha\beta}}{R_{ij}^5} \tag{S6}$$

where $\mathbf{R}_{ij}$ is the vector between transition dipole centers i and j (thus R_{ij} is the distance between centers), and R_{ij}^{α} is the α component of the vector $\mathbf{R}_{ij}$. For the purposes of this model, we assume the transition dipole moments are located at the center of mass of each molecule. The resulting exciton Hamiltonian is block-diagonal, with uncoupled molecular fragment modes on the diagonal and coupling between transition dipole moments on different molecules in the off-diagonal blocks. For a system with two monomers A and B, where A has M_A normal modes and B has M_B normal modes, this Hamiltonian takes the form

$$\mathbf{H} = \begin{pmatrix} \omega_{A_1} & 0 & \cdots & 0 & V_{A_1 B_1} & V_{A_1 B_2} & \cdots & V_{A_1 B_{M_B}} \\ 0 & \omega_{A_2} & \ddots & \vdots & V_{A_2 B_1} & V_{A_2 B_2} & \cdots & V_{A_2 B_{M_B}} \\ \vdots & \ddots & \ddots & 0 & \vdots & \vdots & \ddots & \vdots \\ 0 & \cdots & 0 & \omega_{A_{M_A}} & V_{A_{M_A} B_1} & V_{A_{M_A} B_2} & \cdots & V_{A_{M_A} B_{M_B}} \\ V_{B_1 A_1} & V_{B_1 A_2} & \cdots & V_{B_1 A_{M_A}} & \omega_{B_1} & 0 & \cdots & 0 \\ V_{B_2 A_1} & V_{B_2 A_2} & \cdots & V_{B_2 A_{M_A}} & 0 & \omega_{B_2} & \ddots & \vdots \\ \vdots & \vdots & \ddots & \vdots & \vdots & \ddots & \ddots & 0 \\ V_{B_{M_B} A_1} & V_{B_{M_B} A_2} & \cdots & V_{B_{M_B} A_{M_A}} & 0 & \cdots & 0 & \omega_{B_{M_B}} \end{pmatrix} . \tag{S7}$$

Note that the diagonal blocks are themselves diagonal, as the *ab initio* normal mode analysis diagonalizes the vibrational Hamiltonians for individual monomers (within the harmonic approximation), thus *intramolecular* mode coupling is already accounted for by the *ab initio* calculations.

Diagonalizing the Hamiltonian in Eq. (S7) affords the solutions to an eigenvalue problem that we write as

$$\hat{H}\,|\tilde{\phi}\rangle = \tilde{\omega}\,|\tilde{\phi}\rangle \ . \tag{S8}$$

We take a product *ansatz* for the local-mode wave functions,

$$|\phi\rangle = \prod_{A=1}^{N} |\phi_A\rangle \ , \tag{S9}$$

where the total vibrational wave function the system is treated as a product of N molecular vibrational wave functions. The eigenvectors of the Hamiltonian, $\mathbf{C}$, are coefficients that rotate the basis vectors from the local-mode basis into the exciton basis:

$$|\tilde{\phi}_n\rangle = \sum_{m}^{\text{modes}} C_{nm}\,|\phi_m\rangle \ . \tag{S10}$$

We denote the function in the exciton-site basis with a tilde ($\tilde{\phi}$), omitting the tilde for functions in the local-mode basis.

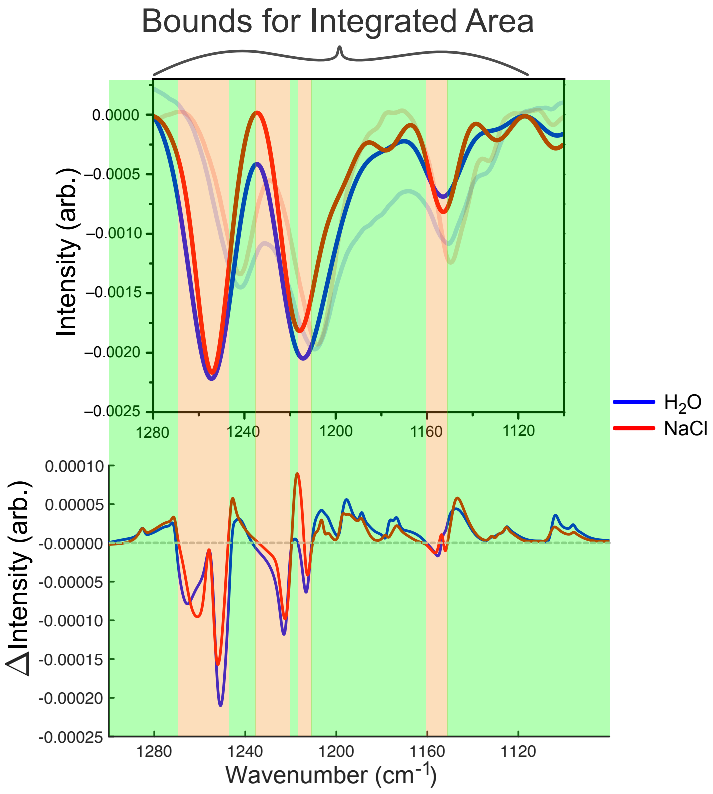

Figure S6: Differences in spectral intensity that are directly caused by transition dipole coupling (bottom) and the associated regions of the exciton spectra impacted by these changes (top). Translucent bars indicate regions of intensity increases (green) and intensity decreases (orange) upon vibrational wavefunction delocalization.

Intensities of the normal modes on isolated molecules can be obtained from their usual relationship to transition dipole moments,

$$I(\omega_i) \propto ||\boldsymbol{\mu}_i||^2 \ . \tag{S11}$$

In order to reflect the coupling of resonant dipole moments in the intensities, we rotate the matrix of isolated molecule transition dipole moments, $\mathbf{M}$, from the local-mode basis into the exciton basis:

$$\tilde{\mathbf{M}} = \mathbf{C}\mathbf{M}\,. \tag{S12}$$

From here, the usual expression can be used with the coupled transition dipole moments to obtain intensities for the interacting normal modes:

$$I(\tilde{\omega}_i) \propto ||\tilde{\boldsymbol{\mu}}_i||^2\,. \tag{S13}$$

The intensity difference spectrum in Fig. S6 shows the difference in signal intensity of the uncoupled PFOA monomers I_0 and the intensity of the fully-coupled system I. Whereas the uncoupled intensities are obtained directly from Eq. (S11), the coupled intensities are those obtained after rotation into the exciton basis [Eq. (S13)]. The results feature intensity increases at shoulder features, decreases near band maxima, and an overall delocalization of the spectral intensity range over a wider region of energies.

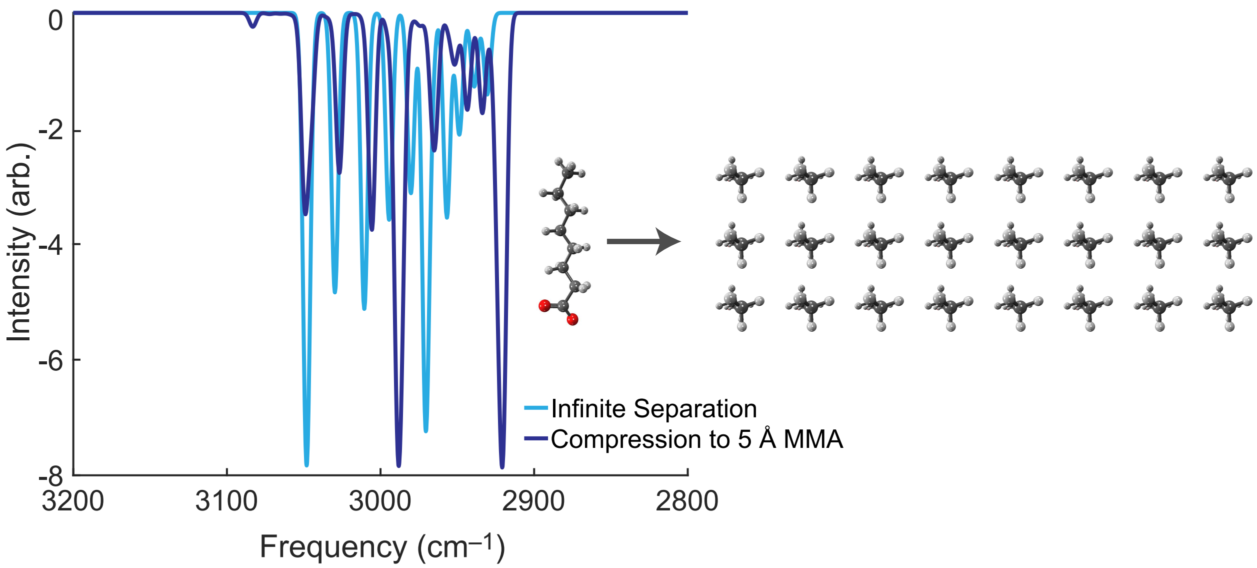

Figure S7: Spectrum of a two-dimensional sheet of octanoic acid (depicted on the right), computed using the *ab initio* vibrational exciton model at two effective mean molecular areas (MMAs). The case of "infinite separation" is modeled by eliminating the off-diagonal coupling blocks of the exciton Hamiltonian, forcing the couplings to zero. The spectrum at 5 Å MMA is modeled with the fully interacting Hamiltonian as a uniformly spaced sheet with 5 Å separation between each molecule.

Finally, we applied the exciton model to a 2D sheet consisting of 24 uniformly spaced octanoic acid molecules, representing a simple model of an ordered monolayer at the air/water interface. Results are shown in Fig. S7 and feature a lowering in the local intensity of the C–H vibrational modes upon compression from an infinitely diffuse monolayer to one corresponding to 5 Å mean molecular area.

S7 Arachidic Acid Monolayer Stability

Arachidic acid (AA) monolayer surface pressure was tracked throughout the infrared reflection-absorption spectroscopic (IRRAS) measurements to ensure that differences in AA C–H peak intensities are due to vibrational excitons and not desorption into the aqueous subphase. The AA surface pressure relative to the initial surface pressure (SP/SP_0) is plotted as a function of time in Fig. S8, and one standard deviation

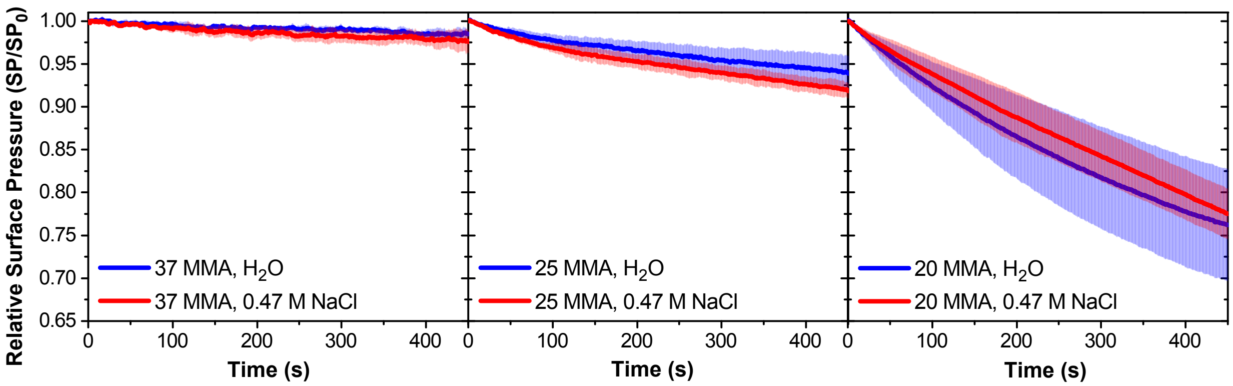

Figure S8: Surface pressure stability of arachidic acid (AA) monolayers on H_2O and 0.47 M NaCl aqueous subphase solutions throughout the time span of an infrared reflection-absorption spectroscopy (IRRAS) experiment. The subphase pH was adjusted to pH 12.5, and 4 μM ethylenediaminetetraacetic acid (EDTA) was added to prevent trace metal binding to the AA carboxylate moieties. Shading represents one standard deviation from the mean.

from the mean is represented by shading. At 37.0 $Å^2$/molecule, the AA surface pressure changes minimally throughout an IRRAS measurement, and the magnitude of change is nearly identical between the water and 0.47 M NaCl aqueous subphase solutions. The decrease in surface pressure becomes slightly greater at 25.0 $Å^2$/molecule such that the relative surface pressure drops to 0.94 ± 0.02 and 0.92 ± 0.01 for the H_2O and NaCl subphase solutions, respectively. If the larger decrease in surface pressure on the 0.47 M NaCl subphase was indicative of AA desorption, then the C–H peak intensity would be smaller in magnitude on the NaCl solution subphase in comparison to the water subphase. However, the opposite is true in that the C–H peak intensity is greater for AA on the NaCl solution subphase, indicating that the peak intensity differences between the aqueous subphase solutions can be attributed to vibrational excitons and not AA desorption. The relative surface pressure measurements for AA at 20.0 $Å^2$/molecule also support this conclusion. While the surface pressure decrease is the largest in magnitude at this particular mean molecular area (MMA), the differences in relative surface pressure are insignificant between the two aqueous subphase solutions (0.75 ± 0.06 on H_2O versus 0.76 ± 0.03 on 0.47 M NaCl).

To affirm the AA monolayer two-dimensional phase assignments and stability at low MMA, Brewster angle microscope (BAM) images of the monolayer were collected throughout the surface pressure-area isotherms. The custom-built BAM setup is equipped with a 1.5 mW He-Ne laser (Meredith Instruments) mounted to a goniometer that positions the laser at the Brewster angle of the air/water interface, 53.1° relative to surface normal. The laser outputs 543 nm light with linear 500:1 polarization, and the p-polarized light is further purified by a Glan-laser calcite polarizer (Thorlabs). The light reflected from the air/water interface is directed through a 10X infinity-corrected super long working distance objective lens (CFI60 TU Plan EPI, Nikon Instruments) followed by a tube lens (MXA22018, Nikon Instruments; focal length 200 mm) to collimate and focus the light at the back-illuminated EM-CCD camera (iXon DV887-BV, Andor Technology USA; 512 × 512 active pixels, 16 μm × 16 μm pixel size). BAM images were cropped from their original size of 8.2 × 8.2 mm using ImageJ software[19] to show the region of highest resolution.

Representative images of the AA monolayer at the MMA values probed spectroscopically are shown in Fig. S9 with the H_2O subphase on the top row and the 0.47 M NaCl subphase on the bottom row. Dark regions of the image correspond to the aqueous subphase, and bright regions correspond to domains, or two dimensional aggregates, of the AA surfactants. In the liquid expanded (LE) phase at 55.5 $Å^2$/molecule, the AA surfactants have not yet aggregated into domains; thus the images are dark on both subphase solutions. Surfactant domains form in the LE - tilted condensed (TC) coexistence region, and the domain shapes differ significantly between the aqueous subphases at 37.0 $Å^2$/molecule. AA on the H_2O subphase forms vine-like domains, whereas the NaCl solution induces small, asymmetrical AA domains. Further compression

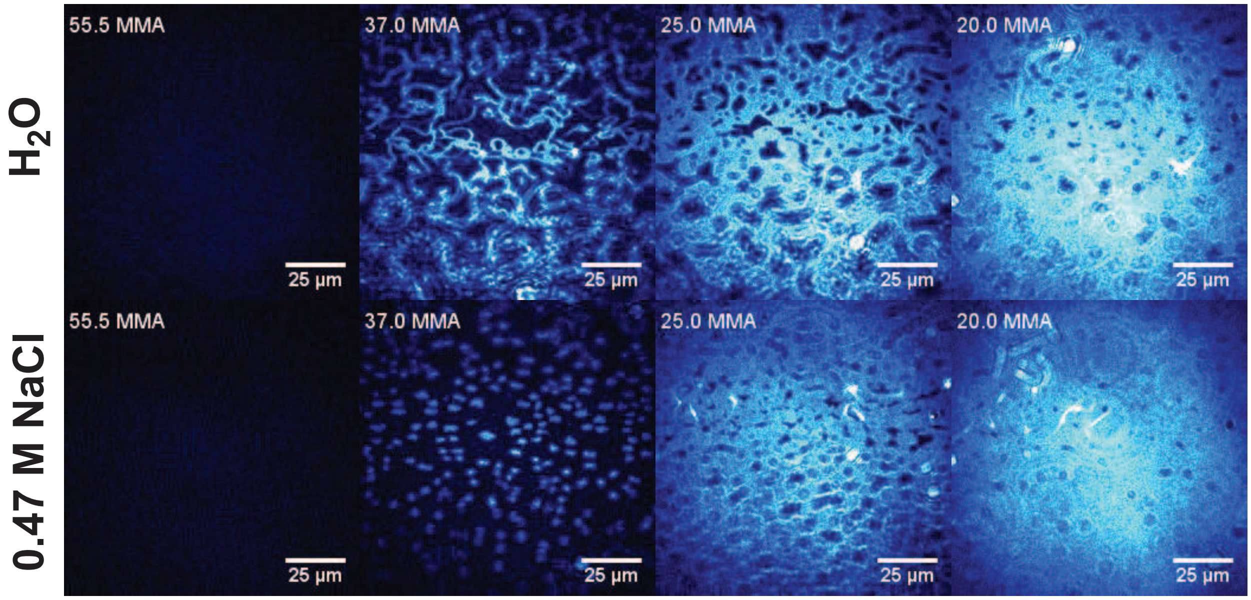

Figure S9: Brewster angle microscopy (BAM) images of arachidic acid (AA) monolayers on H_2O (top row) and on 0.47 M NaCl (bottom row). The aqueous subphase solutions were adjusted to pH 12.5, and 4 μM ethylenediaminetetraacetic acid (EDTA) was added to prevent trace metal binding to the AA carboxylate moieties.

to 25.0 Å^2/molecule causes the domains to merge. AA on H_2O forms brighter merged domain structures and larger dark regions than AA on 0.47 M NaCl, suggesting that the surfactants aggregate more closely to one another on H_2O. The trend is still apparent in the TC phase at 20 Å^2/molecule, although the AA domains have largely merged into one sheet. The brighter domain structures and greater dark space within the BAM images of AA on H_2O suggest that AA surfactants aggregate more tightly together, meaning that vibrational excitons between the surfactants are more significant for AA on H_2O than for AA on the NaCl solution subphase.

References

[1] Y. Shao, Z. Gan, E. Epifanovsky, A. T. B. Gilbert, M. Wormit, J. Kussmann, A. W. Lange, A. Behn, J. Deng, X. Feng, D. Ghosh, M. Goldey, P. R. Horn, L. D. Jacobson, I. Kaliman, R. Z. Khaliullin, T. Kús, A. Landau, J. Liu, E. I. Proynov, Y. M. Rhee, R. M. Richard, M. A. Rohrdanz, R. P. Steele, E. J. Sundstrom, H. L. Woodcock III, P. M. Zimmerman, D. Zuev, B. Albrecht, E. Alguire, B. Austin, G. J. O. Beran, Y. A. Bernard, E. Berquist, K. Brandhorst, K. B. Bravaya, S. T. Brown, D. Casanova, C.-M. Chang, Y. Chen, S. H. Chien, K. D. Closser, D. L. Crittenden, M. Diedenhofen, R. A. DiStasio Jr., H. Do, A. D. Dutoi, R. G. Edgar, S. Fatehi, L. Fusti-Molnar, A. Ghysels, A. Golubeva-Zadorozhnaya, J. Gomes, M. W. D. Hanson-Heine, P. H. P. Harbach, A. W. Hauser, E. G. Hohenstein, Z. C. Holden, T.-C. Jagau, H. Ji, B. Kaduk, K. Khistyaev, J. Kim, J. Kim, R. A. King, P. Klunzinger, D. Kosenkov, T. Kowalczyk, C. M. Krauter, K. U. Lao, A. Laurent, K. V. Lawler, S. V. Levchenko, C. Y. Lin, F. Liu, E. Livshits, R. C. Lochan, A. Luenser, P. Manohar, S. F. Manzer, S.-P. Mao, N. Mardirossian, A. V. Marenich, S. A. Maurer, N. J. Mayhall, C. M. Oana, R. Olivares-Amaya, D. P. O'Neill, J. A. Parkhill, T. M. Perrine, R. Peverati, P. A. Pieniazek, A. Prociuk, D. R. Rehn, E. Rosta, N. J. Russ, N. Sergueev, S. M. Sharada, S. Sharma, D. W. Small, A. Sodt, T. Stein, D. Stück, Y.-C. Su, A. J. W. Thom, T. Tsuchimochi, L. Vogt, O. Vydrov, T. Wang, M. A. Watson, J. Wenzel, A. White, C. F. Williams, V. Vanovschi, S. Yeganeh, S. R. Yost, Z.-Q. You, I. Y. Zhang, X. Zhang, Y. Zhao, B. R. Brooks, G. K. L. Chan, D. M. Chipman, C. J. Cramer, W. A. Goddard III, M. S. Gordon, W. J. Hehre, A. Klamt, H. F. Schaefer III, M. W. Schmidt, C. D. Sherrill, D. G. Truhlar, A. Warshel, X. Xu, A. Aspuru-Guzik, R. Baer, A. T. Bell, N. A. Besley, J.-D. Chai, A. Dreuw, B. D. Dunietz, T. R. Furlani, S. R. Gwaltney, C.-P. Hsu, Y. Jung, J. Kong, D. S. Lambrecht, W. Liang, C. Ochsenfeld, V. A. Rassolov, L. V. Slipchenko, J. E. Subotnik, T. Van Voorhis, J. M. Herbert, A. I. Krylov, P. M. W. Gill, and M. Head-Gordon, Advances in molecular quantum chemistry contained in the Q-Chem 4 program package, *Mol. Phys.*, 2015, **113**, 184–215.

[2] C. Y. Lin, M. W. George, and P. M. W. Gill, EDF2: A density functional for predicting molecular vibrational frequencies, *Aust. J. Chem.*, 2004, **57**, 365–370.

[3] J. P. Merrick, D. Moran, and L. Radom, An evaluation of harmonic vibrational frequency scaling factors, *J. Phys. Chem. A*, 2007, **111**, 11683–11700.

[4] L. Martínez-Balbuena, A. Arteaga-Jiménez, E. Hernández-Zapata, and C. Márquez-Beltrán, Applicability of the Gibbs adsorption isotherm to the analysis of experimental surface-tension data for ionic and nonionic surfactants, *Adv. Colloid Interfac.*, 2017, **247**, 178–184.

[5] J. Costanza, M. Arshadi, L. M. Abriola, and K. D. Pennell, Accumulation of PFOA and PFOS at the air–water interface, *Environ. Sci. Technol. Lett.*, 2019, **6**, 487–491. Erratum: *Environ. Sci. Technol. Lett.*, 2020, **7**, 446.

[6] I. Langmuir, The constitution and fundamental properties of solids and liquids. II. Liquids, *J. Am. Chem. Soc.*, 1917, **39**, 1848–1906.

[7] K. Lunkenheimer, D. Prescher, R. Hirte, and K. Geggel, Adsorption properties of surface chemically pure sodium perfluoro-*n*-alkanoates at the air/water interface: Counterion effects within homologous series of 1:1 ionic surfactants, *Langmuir*, 2015, **31**, 970–981.

[8] A. J. Grooms, J. F. Neal, K. C. Ng, W. Zhao, A. H. Flood, and H. C. Allen, Thermodynamic signatures of the origin of *anti*-Hofmeister selectivity for phosphate at aqueous interfaces, *J. Phys. Chem. A*, 2020, **124**, 5621–5630.

[9] H. Nakahara, S. Nakamura, H. Kawasaki, and O. Shibata, Properties of two-component Langmuir monolayer of single chain perfluorinated carboxylic acids with dipalmitoylphosphatidylcholine (DPPC), *Colloid Surface B*, 2005, **41**, 285–298.

[10] T. Shimoaka, Y. Tanaka, N. Shioya, K. Morita, M. Sonoyama, H. Amii, T. Takagi, T. Kanamori, and T. Hasegawa, Surface properties of a single perfluoroalkyl group on water surfaces studied by surface potential measurements, *J. Colloid Interf. Sci.*, 2016, **483**, 353–359.

[11] H. D. Downing and D. Williams, Optical constants of water in the infrared, *J. Geophys. Res.*, 1975, **80**, 1656–1661.

[12] J. C. Wu, G. Chattree, and P. Ren, Automation of AMOEBA polarizable force field parameterization for small molecules, *Theor. Chem. Acc.*, 2012, **131**, 1138.

[13] P. Ren, C. Wu, and J. W. Ponder, Polarizable atomic multipole-based molecular mechanics for organic molecules, *J. Chem. Theory Comput.*, 2011, **7**, 3143–3161.

[14] A. J. Stone, Distributed multipole analysis: Stability for large basis sets, *J. Chem. Theory Comput.*, 2005, **1**, 1128–1132.

[15] R. M. Parrish, L. A. Burns, D. G. A. Smith, A. C. Simmonett, A. E. DePrince III, E. G. Hohenstein, U. Bozkaya, A. Y. Sokolov, R. Di Remigio, R. M. Richard, J. F. Gonthier, A. M. James, H. R. McAlexander, A. Kumar, M. Saitow, X. Wang, B. P. Pritchard, P. Verma, H. F. Schaefer III, K. Patkowski, R. A. King, E. F. Valeev, F. A. Evangelista, J. M. Turney, T. D. Crawford, and C. D. Sherrill, Psi4 1.1: An open-source electronic structure program emphasizing automation, advanced libraries, and interoperability, *J. Chem. Theory Comput.*, 2017, **13**, 3185–3197.

[16] A. J. Stone and M. Alderton, Distributed multipole analysis: Methods and applications, *Mol. Phys.*, 1985, **56**, 1047–1064.

[17] J. A. Rackers, Z. Wang, C. Lu, M. L. Laury, L. Lagardère, M. J. Schnieders, J.-P. Piquemal, P. Ren, and J. W. Ponder, Tinker 8: Software tools for molecular design, *J. Chem. Theory Comput.*, 2018, **14**, 5273–5289.

[18] J. Wang, R. M. Wolf, J. W. Caldwell, P. A. Kollman, and D. A. Case, Development and testing of a general Amber force field, *J. Comput. Chem.*, 2004, **25**, 1157–1174.

[19] C. A. Schneider, W. S. Rasband, and K. W. Eliceiri, NIH Image to ImageJ: 25 years of image analysis, *Nat. Methods*, 2012, **9**, 671–675.
